# Dimensionless parameter predicts bacterial prodrug success

**DOI:** 10.15252/msb.202110495

**Published:** 2022-01-10

**Authors:** Brandon Alexander Holt, McKenzie Tuttle, Yilin Xu, Melanie Su, Joachim J Røise, Xioajian Wang, Niren Murthy, Gabriel A Kwong

**Affiliations:** ^1^ Wallace H. Coulter Department of Biomedical Engineering Georgia Tech College of Engineering and Emory School of Medicine Atlanta GA USA; ^2^ Department of Bioengineering Innovative Genomics Institute University of California Berkeley CA USA; ^3^ Institute of Advanced Synthesis School of Chemistry and Molecular Engineering Nanjing Tech University Nanjing China; ^4^ Parker H. Petit Institute of Bioengineering and Bioscience Atlanta GA USA; ^5^ Institute for Electronics and Nanotechnology Georgia Tech Atlanta GA USA; ^6^ Integrated Cancer Research Center Georgia Tech Atlanta GA USA; ^7^ Georgia ImmunoEngineering Consortium Georgia Tech and Emory University Atlanta GA USA; ^8^ Emory School of Medicine Atlanta GA USA; ^9^ Emory Winship Cancer Institute Atlanta GA USA

**Keywords:** antibiotic failure, bacteria, enzymes, minimum inhibitory concentration, prodrugs, Microbiology, Virology & Host Pathogen Interaction, Pharmacology & Drug Discovery

## Abstract

Understanding mechanisms of antibiotic failure is foundational to combating the growing threat of multidrug‐resistant bacteria. Prodrugs—which are converted into a pharmacologically active compound after administration—represent a growing class of therapeutics for treating bacterial infections but are understudied in the context of antibiotic failure. We hypothesize that strategies that rely on pathogen‐specific pathways for prodrug conversion are susceptible to competing rates of prodrug activation and bacterial replication, which could lead to treatment escape and failure. Here, we construct a mathematical model of prodrug kinetics to predict rate‐dependent conditions under which bacteria escape prodrug treatment. From this model, we derive a dimensionless parameter we call the Bacterial Advantage Heuristic (*BAH*) that predicts the transition between prodrug escape and successful treatment across a range of time scales (1–10^4^ h), bacterial carrying capacities (5 × 10^4^–10^5^ CFU/µl), and Michaelis constants (*K_M_* = 0.747–7.47 mM). To verify these predictions *in vitro*, we use two models of bacteria‐prodrug competition: (i) an antimicrobial peptide hairpin that is enzymatically activated by bacterial surface proteases and (ii) a thiomaltose‐conjugated trimethoprim that is internalized by bacterial maltodextrin transporters and hydrolyzed by free thiols. We observe that prodrug failure occurs at *BAH* values above the same critical threshold predicted by the model. Furthermore, we demonstrate two examples of how failing prodrugs can be rescued by decreasing the *BAH* below the critical threshold via (i) substrate design and (ii) nutrient control. We envision such dimensionless parameters serving as supportive pharmacokinetic quantities that guide the design and administration of prodrug therapeutics.

## Graphical abstract

Analyses of potential prodrug failure mechanisms show that prodrugs that rely on pathogen‐specific pathways for conversion are susceptible to competing rates of prodrug activation and bacterial replication, which could lead to prodrug treatment escape and failure.

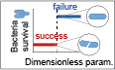

## Introduction

The rise of multidrug‐resistant bacteria coupled with the lack of newly developed antibiotic treatment strategies has created a serious public health threat (Baquero *et al*, 2008; Gullberg *et al*, 2011). Antibiotic success is markedly improved by proper titration of drugs, as overdosing leads to off‐target toxicity and underdosing increases the likelihood of pathogens developing resistance (Opatowski *et al*, 2010). However, optimal drug doses are difficult to achieve over the course of treatment because infection burden changes dynamically over time, creating a moving target (Opatowski *et al*, 2010; Iizumi *et al*, 2017). Prodrugs, which represent ~10% of all FDA‐approved drugs in the last decade (Rautio *et al*, 2018a), are a promising solution because they present multiple strategies for reviving existing or previously discarded antibiotics (Jubeh *et al*, 2020); these strategies include increasing bioavailability and solubility, reducing off‐target effects, or targeting bacteria‐specific enzymes. For example, a prodrug of ciprofloxacin was developed that reduced off‐target toxicity while selectively targeting bacteria expressing ß‐lactamase (i.e., a resistance enzyme that degrades ß‐lactam antibiotics) (Evans *et al*, 2019). Prodrug forms of Triclosan (Howse *et al*, 2019), Carvacrol (Marinelli *et al*, 2019), and multiple nucleoside derivatives (Negrya *et al*, 2020) were developed which increased solubility while maintaining antimicrobial efficacy. In this work we focus specifically on antibiotic prodrugs, a subset of prodrugs that includes compounds such as ganciclovir (Al‐Badr & Ajarim, 2018) and isoniazid (Metcalfe *et al*, 2008), which are administered as biologically inactive forms and are enzymatically activated into their parent form by the pathogen.

Despite their growing importance, prodrugs are critically understudied in the context of potential failure mechanisms that may arise during treatment. Currently, failure mechanisms fall into three distinct categories—resistance, persistence, and tolerance—which are characterized by the change in drug concentration and exposure time required to kill bacteria. For example, resistance is characterized by genetic mutations or phenotypic changes which result in bacteria requiring significantly higher concentrations of antibiotic (minimum inhibitory concentration, MIC) to be lethal. In contrast, bacteria exhibiting tolerance or persistence require increased drug exposure time (minimum duration for killing, MDK) (Brauner *et al*, 2016). However, prodrug activation introduces an additional reaction step; this two‐step (Jain *et al*, 2009) mechanism (i.e., activation + killing) creates variability in the concentration and duration required for killing, which suggests that classification metrics such as MIC and MDK may not map directly from parent to prodrugs. Computational studies have shown that the prodrug activation step results in distinct differences in kinetics between parent and prodrug forms of the same compound (Jackson *et al*, 2000; Murphy *et al*, 2011; Cho & Yoon, 2018). For example, agent‐based simulations revealed that the rate of prodrug activation (i.e., catalytic efficiency, *k_cat_*/*K_M_*) had a strong effect on the MIC of each compound (Murphy *et al*, 2011). Furthermore, empirical studies have found there to be differences in MIC between parent and prodrug forms, as well as higher variance in prodrug MIC across bacterial strains, relative to the parent drug (Wang *et al*, 2018; Evans *et al*, 2019; Yang *et al*, 2021).

Here, we develop a mathematical model of bacteria‐prodrug systems to probe failure mechanisms and identify a prodrug‐specific metric distinct from MIC to classify failure. We apply our model to two *in vitro* systems: (i) a prodrug of a cationic antimicrobial peptide (AMP) polyarginine (R_9_) targeting DH5α *E. coli* and (ii) a prodrug of trimethoprim (TMP) targeting UTI89 *E. coli*. The polyarginine AMP is formulated as a prodrug by charge complexation with anionic peptides connected by a modular protease‐cleavable linker substrate (Olson *et al*, 2009, 2010; Forde *et al*, 2014). We design the linker substrate to be cleaved by *E. coli* protease OmpT, such that increasing concentrations of bacteria activate higher concentrations of free AMP. The trimethoprim prodrug comprises thiomaltose conjugated to trimethoprim via a self‐immolative disulfide linker, which releases TMP‐OH upon cleavage by free thiols inside bacterial cells without affecting the toxicity of the drug (Wang *et al*, 2018). Free TMP‐OH then kills bacteria by inhibiting tetrahydrofolic acid synthesis, which is a necessary cofactor for thymidine, purine, and bacterial DNA synthesis (Masters *et al*, 2003).

In both prodrug systems, we observe experimental conditions where (i) bacteria proliferate in the presence of active drug by consistently outpacing prodrug activation at all stages of growth (i.e., log phase, stationary phase) as well as (ii) conditions where bacteria are successfully treated. To create a metric that predicts the transition between these treatment outcomes we identify a dimensionless parameter, the Bacterial Advantage Heuristic (*BAH*). Dimensionless parameters characterize physical systems across a wide range of scales (e.g., time, length, temperature, etc.); for example, the dimensionless Reynolds number (*Re*) predicts the transition from laminar (low *Re*) to turbulent (high *Re*) fluid flow (Batchelor, 2000). Similarly, we show that the *BAH* predicts the transition from successful treatment (low *BAH*) to prodrug escape (high *BAH*) with high accuracy (AUROC = 1.00, *n* = 9) across a range of environmental conditions (e.g., temperatures, nutrient levels). We envision that such a dimensionless parameter may be useful for predicting prodrug success across a broad range of treatment conditions which may extend to clinical use cases. These quantitative insights may inform future drug design and treatment protocols for improving the impact of prodrugs in combatting antibiotic resistance.

## Results

### A computational model of bacteria‐prodrug activation kinetics

**Figure 1:**
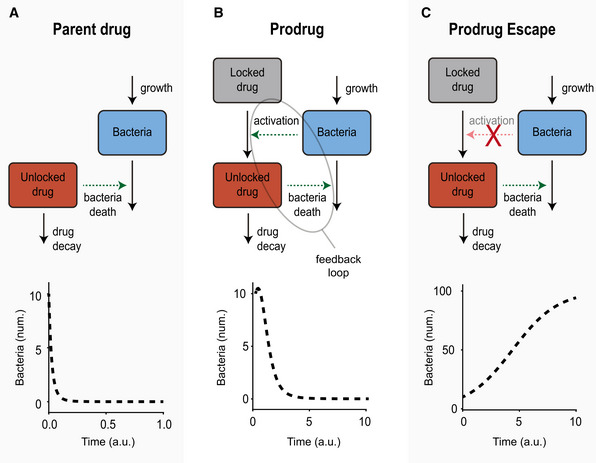
A computational model of bacteria‐prodrug activation kinetics Standard model of bacteria‐drug kinetics (i.e., the parent drug model). (Top) General mass action schematic of bacteria population (blue box) versus the unlocked drug population (red box). The unlocked drug increases the rate of bacterial death (i.e., green arrow labeled "bacteria death"). (Bottom) Computational results of the parent drug model, plotting the living bacteria population over time.Our model of bacteria‐prodrug kinetics (i.e., the prodrug model). (Top) General mass action schematic of the locked drug (gray box), which is activated (green arrow labeled "activation") by the bacteria (blue box) and converted into the unlocked drug (red box). (Bottom) Computational results of the prodrug model, plotting the decay in the living bacteria population over time.The model of prodrug escape, (Top) where the activation rate of the locked drug is lowered significantly (red arrow with "X"). (Bottom) Computational results of the prodrug escape model, plotting the growth in the number of living bacteria over time. In all plots, bacteria are plotted as number of cells (num., y‐axis), and time is plotted in arbitrary units (a.u., x‐axis).

Models of parent drug kinetics (Nielsen *et al*, 2007, 2011; Nguyen *et al*, 2014) generally do not have feedback loops, meaning the drug population affects the bacterial population (i.e., bacterial death, green arrow), but the bacteria do not influence the drug population (Fig 1A). By comparison, models of prodrug kinetics do have a feedback loop as bacteria determine the growth of the drug population (i.e., activation, green arrow), which kills bacteria. We hypothesized that this feedback loop creates competition between the rate of prodrug activation and the rate of bacterial death (Fig 1B). Interestingly, this may enable possibilities where bacteria escape prodrug treatment; for example, when the activation rate is decreased to a near‐zero value (i.e., activation, red arrow with "X"), then the rate of bacterial death is minimized, allowing the bacteria population to grow uncontrolled (Fig 1C). To quantitatively understand the rate‐competition between bacteria and prodrugs, we built a mathematical compartment model using a system of nonlinear ordinary differential equations (ODE). In this system, the three dynamic populations were the Bacteria, *B*, the Locked drug (i.e., prodrug), *L*, and the Unlocked drug (i.e., parent drug), *U*, for which we formulated governing ODEs by considering the system parameters that affect population change over time. We modeled the bacteria population, *B,* as increasing the rate of prodrug conversion (i.e., *L* to *U*) and the unlocked drug population *U* as increasing the rate of bacterial death (Fig 1B). To account for the fact that bacterial growth rate, *r*, slows down as environmental resources become limiting (i.e., carrying capacity, *B_max_*), we used a logistic growth model (Fujikawa *et al*, 2003), which produces an S‐shaped curve and has been used extensively in biology to study population expansion (Verhulst, 1845) and tumor growth (Atuegwu *et al*, 2013). In contrast, we model the rate of bacterial death as proportional to the concentration of unlocked drug, *U*, and the concentration of Bacteria, *B,* according to a proportionality rate constant, *a*, which represents the bacterial death rate constant (Equation 1.1, Table EV2).

(1.1) $$\frac{\mathrm{dB}}{\mathrm{dt}}=rB\left(1‐\frac{B}{B_{\max}}\right)‐aBU$$

To model the rate of activation of locked drugs, *L*, we applied Michaelis–Menten (MM) kinetics (Menten & Michaelis, 1913), where the rate of substrate activation is determined by the catalytic rate of the reaction, *k_cat_*, and the half‐maximal substrate concentration, *K_M_*. Here, we modeled the locked drug as the substrate and the unlocked drug as the product; therefore, we modeled the bacteria as the enzymatic population because the bacteria convert locked drug (i.e., substrate) to unlocked drug (i.e., product) (Equation 1.2). To apply MM kinetics, we assumed our system constituted a well‐mixed solution of freely diffusing substrates (i.e., locked drug) in large excess relative to the number of bacteria enzymes, which were valid assumptions for our downstream studies since prodrugs were present at concentrations ~10^2^ micromolar in an aqueous environment relative to a maximum bacterial concentration of ~10^−6^ micromolar. Because the total amount of drug is conserved, we defined the MM activation rate of unlocked drug, *U*, as opposite of the degradation rate of locked drug *L*. We further included a term to account for the loss of unlocked drug according to a proportionality constant, *b*, which represents the drug decay rate constant (Equation 1.3, Table EV2).

(1.2) $$\frac{\mathrm{dL}}{\mathrm{dt}}=‐k_{\mathrm{cat}}B\frac{L}{K_{m}+L}$$

(1.3) $$\frac{\mathrm{dU}}{\mathrm{dt}}=k_{\mathrm{cat}}B\frac{L}{K_{m}+L}‐bBU$$

We performed a linear stability analysis on this system of differential equations and found that there are two unique steady‐state solutions. The analysis revealed that the solution that includes the result *B_s.s_* = *B_max_* is stable, whereas the other solution (i.e., *B_s.s_* = 0) is unstable (Supplementary analysis).

### Predicting prodrug success with a dimensionless parameter

We hypothesized that the primary mechanism controlling prodrug success or escape is tied to the competition between bacterial growth and prodrug activation (Fig 1B and C). Therefore, we chose to focus our studies on a dimensionless parameter that represents the competing ratio of growth rate (*r*) divided by prodrug activation rate (*k_cat_*). We defined this dimensionless parameter as the Bacterial Advantage Heuristic (*BAH*) and we calculated the log of this quantity since bacterial quantities span across several orders of magnitude (Equation 2.1).

(2.1) $$BAH=\log_{10}\left(\frac{r}{k_{\mathrm{cat}}}\right)$$

**Figure 2:**
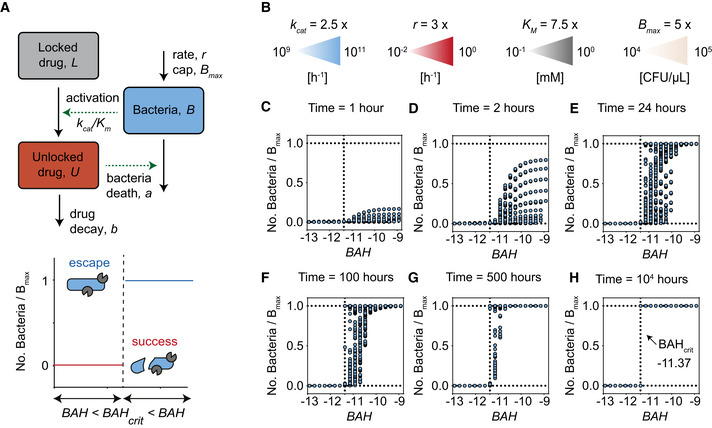
Predicting prodrug success with a dimensionless parameter A(Top) Schematic of general mass action model of bacteria‐activated prodrug therapies. Each arrow represents a biological process and is labeled with the system parameters related to that process. Solid black arrows represent growth or decay expressions, and green arrows represent when one population influences the growth or decay expression of another population (e.g., bacteria activate locked drug population). (Bottom) Schematic plot of predicted relationship between *BAH* value and final number of living bacteria (normalized by *B_max_*). Conditions where bacteria numbers reach carrying capacity (blue line) are called prodrug escape conditions. Conditions where bacteria numbers reach 0 (red line) are called prodrug success conditions. The vertical dashed line represents the critical *BAH* value (i.e., *BAH_crit_*), the point at which the condition switches from prodrug success to prodrug escape. Blue ovals with gray pac mans represent bacteria either surviving (whole), or dying (fragmented).BLegend showing the range of parameters used in the computational simulations. The range of each parameter is calculated by multiplying a constant base value (top number) with logarithmically spaced values between two powers of 10 (left and right values). The units are displayed below in brackets.C–HScatter plots showing the number of bacteria surviving (No. Bacteria) at a particular time point (time point in graph title), normalized by the carrying capacity (*B_max_*) (y‐axis), versus the *BAH* value for that system (x‐axis). Horizontal dashed lines represent upper and lower limits to bacteria number (i.e., 0 = all bacteria dead, 1 = bacteria reached carrying capacity). Vertical dashed line represents computationally derived critical *BAH* value (i.e., *BAH_crit_*) at which systems switch prodrug success (0) to prodrug escape (1).

In this form, the *BAH* is larger when the rate of bacterial growth (*r*) increases relative to the rate of prodrug activation (*k_cat_*); according to our hypothesis, conditions with larger *BAH* values would yield an increased probability of prodrug escape. To verify this computationally, we sought to determine the critical *BAH* value (*BAH_crit_*) that distinguishes prodrug escape from prodrug success (Fig 2A). Using our mathematical model, we simulated > 2,500 prodrug treatment conditions covering a range of values for *r* (3 × 10^−2^–10^0^ h^−1^), *B_max_* (5 × 10^4^–10^5^ CFU/µl), *k_cat_* (2.5 × 10^9^–10^11^ h^−1^), and *K_m_* (0.747–7.47 mM) each spanning at least an order of magnitude (Fig 2B). We fixed the bacteria death rate constant, *a*, and the drug decay rate constant, *b*, because these parameters are closely linked to the identity of the bacterial strain and prodrug formulation, meaning that changing these values would reflect an entirely different treatment scenario altogether (i.e., different bacteria species and/or drug). We plotted the number of surviving bacteria at various time points divided by the bacterial carrying capacity of the system to normalize against different *B_max_* values. We observed that at early time points (i.e., *t* < 24 h) conditions with smaller *BAH* certainly reached a final value of 0, whereas conditions with larger *BAH* resulted in a distribution between 0 and 1 (Fig 2C–E). However, the model showed that as the system moves toward steady‐state (i.e., *t* > 24 h), conditions with high *BAH* approach a final value of 1, revealing a critical value of *BAH* (*BAH_crit_* ~ −11.37) (Fig 2F–H). In other words, the model predicts that for any environmental condition that produces a *BAH* > *BAH_crit_*, the bacteria will ultimately escape the prodrug treatment, and for *BAH* < *BAH_crit_*, the bacteria will die (i.e., prodrug success). These dimensionless parameters may be important for guiding the successful design and administration of prodrug therapies, which can be improved by optimizing fundamental pharmacokinetic parameters.

### A bacteria‐activatable AMP prodrug targets *E. coli* protease OmpT

**Figure 3:**
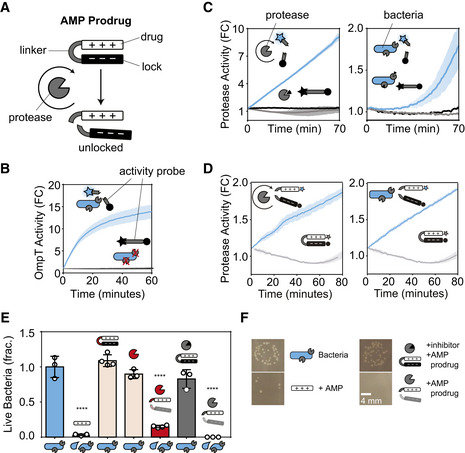
A bacteria‐activatable prodrug targets *E. coli* protease OmpT A cationic AMP drug (R_9_, white rectangle) locked by an anionic peptide lock (E_13_, black rectangle) with a protease‐cleavable linker (RRS|RRV, gray u‐shape) is activated by OmpT protease (gray pac man) activity.Cleavage assay measuring the activity of OmpT expressed on the surface of parent strain *E. coli* K‐12 BW25113 (OmpT‐positive; blue bacteria with gray pac mans, top) as well as OmpT genetic knockouts (OmpT‐negative; blue bacteria with red X's). Activity is measured using an activity probe, comprising a linear peptide substrate (gray bar) with a fluorophore (blue/gray star) and quencher (black circle) on either end.Cleavage assay using activity probes to measure the activity of recombinant OmpT (gray protease) (left) and OmpT expressed on the surface of *E. coli* bacteria (right), plotted as the blue lines on the graph. Negative control samples contain the inhibitor aprotinin (black triangle) or linker substrates alone (i.e., no proteases added), which are plotted as black and gray lines, respectively.Cleavage assay measuring the activity of recombinant OmpT (left) or OmpT expressed on the surface of *E. coli* (right) against fluorescently labeled hairpin prodrugs (blue lines) or hairpin prodrugs only control (gray lines).Bacteria viability assay quantifying drug toxicity relative to untreated bacteria control (blue bar). Positive control for AMP toxicity (black bar). Negative control for locked AMP or TEV protease alone (tan bars). Positive control for TEV protease (red pac man) with locked AMP (substrate: ENLYFQ|G, specific to TEV protease) (red bar). Negative control for locked AMP (substrate: RRSRRV, specific to OmpT) with OmpT inhibitor, aprotinin (gray bar). Experimental condition of bacteria treated with locked AMP activated by natively expressed OmpT (far right bar). All values normalized and compared to bacteria only control via one‐way ANOVA, and are plotted as fraction of bacteria only control (y‐axis, fraction). Error bars represent standard deviation (*n* = 3–4 biological replicates). ****P < 0.0001.Representative images of bacterial plates used to quantify viability with schematic legend (scale bar = 4 mm). Data information: For all line graphs, shaded regions represent standard deviation (*n* = 3 biological replicates). All cleavage assays (i.e., line graphs) plotted as fold change (FC) in relative fluorescence units (RFU) from initial time point.

To validate the predictions of our mathematical model with an *in vitro* bacteria‐activated prodrug system, we synthesized a protease‐activated AMP prodrug. This AMP prodrug comprised cationic (polyarginine, R_9_) antimicrobial peptides (AMP) in charge complexation with anionic peptide locks (polyglutamic acid, E_13_) by a linker peptide (RRS|RRV) specific for the ubiquitous bacterial protease OmpT (Olson *et al*, 2009; preprint: Holt *et al*, 2019). Upon proteolytic cleavage of the linker, the hairpin prodrug is unlocked to release free AMP (Fig 3A). To measure OmpT activity, we synthesized an activity probe (McCarter *et al*, 2004; Kwong *et al*, 2013, 2021; Holt *et al*, 2017, 2018; Mac *et al*, 2019; Zhuang *et al*, 2019) with free linker peptides containing a fluorophore‐quencher pair, which produced an increase in fluorescence upon proteolytic cleavage. To demonstrate linker specificity for OmpT, we incubated the activity probe with OmpT genetic knockout bacteria, as well as the parent background strain (*E. coli* K‐12 BW25113), and only observed activity in samples incubated with the parent strain (i.e., OmpT‐positive) (Fig 3B). We also observed no activity in samples containing the serine protease inhibitor, Aprotinin, which inhibits OmpT when present in micromolar concentrations (Brannon *et al*, 2015), confirming the linker specificity for OmpT (Fig 3C). We observed a similar cleavage activity using this linker substrate when fully integrated into hairpin AMP drug‐lock complexes, confirming that linker presentation within a constrained conformational state did not significantly affect cleavage activity by OmpT (Fig 3D). To measure the cytotoxicity of the unlocked drug, we dosed bacteria with free AMP and observed significant reduction in colonies compared to untreated controls (blue bars) (Fig 3E and F). To confirm prodrug specificity, we synthesized AMP drug‐lock complexes using linker peptides specific for OmpT or tobacco etch virus (TEV) protease, which exhibits orthogonal protease specificity (Kapust *et al*, 2001). We observed elimination of bacteria only in samples containing OmpT‐specific AMP prodrug (gray bars) or samples treated with both TEV and TEV‐specific AMP prodrug (red bars). All control samples containing either TEV‐specific prodrug alone or Aprotinin inhibitor did not significantly reduce bacteria load (Fig 3E and F, Table EV1). These results showed that AMP drug‐lock complexes are inert and lack cytotoxic activity until activation by protease activity.

### Validating the model and predicting prodrug success with an AMP Prodrug and DH5α *E. coli*

**Figure 4:**
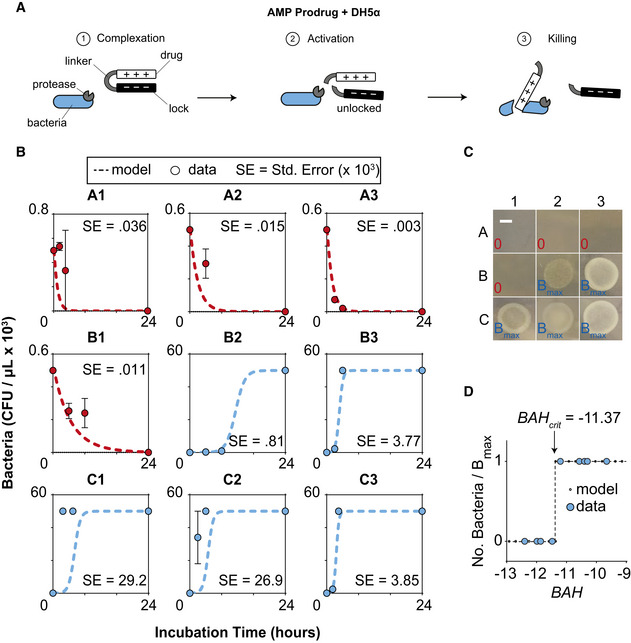
Validating the model and predicting prodrug success with *E. coli* DH5a and AMP Prodrug Graphical representation of the activation of an AMP prodrug (black and white U‐shape) by a membrane protease (gray pac man, OmpT) on a bacteria (blue). The AMP prodrug first (step 1) complexes with a membrane‐bound protease. The protease enzymatically cleaves the AMP prodrug, thereby, activating the prodrug (step 2). The freed AMP kills the bacteria by intercalating with the membrane causing fatal damage (step 3).Validating the model with serial CFU measurements (red and blue dots; *n* = 3 biological replicates, error bars SEM) and ODE model simulations of nine conditions (A1–3, B1–3, C1–3) given extracted growth rate and enzyme kinetics parameter values. Standard error (SE) represents the difference between model predictions and experimental observation.Agar plates taken at endpoint plotting the resulting bacterial growth for nine environmental conditions (A1–C3, scale bar = 4 mm).Plotting the resulting endpoint bacterial growth for each of the nine conditions (plotted as number of bacteria normalized by carrying capacity *B_max_*) versus the calculated *BAH* number (blue dots). This is compared against the values predicted by the model (black dots). The critical *BAH* value that separates prodrug success conditions from prodrug failure conditions is represented by the vertical dashed line (i.e., *BAH_crit_* = −11.37).

**Figure EV1:**
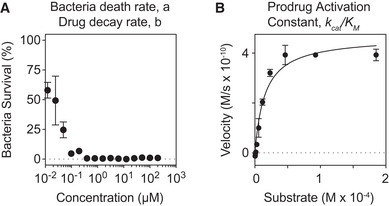
Measuring kinetic parameters for the AMP Prodrug model Drug dosing experiment plotting percent of bacteria surviving (CFU count divided by number of CFUs in no drug control) versus the concentration of the parent drug (i.e., free AMP, polyarginine). Experiment was taken at a short time interval (*t* < 10 min) so we could calculate the number of bacteria and drug copies consumed in each killing reaction. This calculation helps us to estimate the parameters (1) bacterial death rate, *a*, and (2) drug decay rate, *b* for the AMP prodrug system.Michaelis–Menten experiment with recombinant OmpT and the AMP prodrug linker substrate. At various concentrations of substrate, we measured the initial velocity (substrates cleaved over time) of the reaction (black dots). Then, we fit the Michaelis–Menten equation to these results (black line) to calculate the *k_cat_* and *K_M_* values for this enzyme–substrate pair. Data information: Error bars are plotted as standard deviation, *n* = 3 biological replicates.

**Figure EV2:**
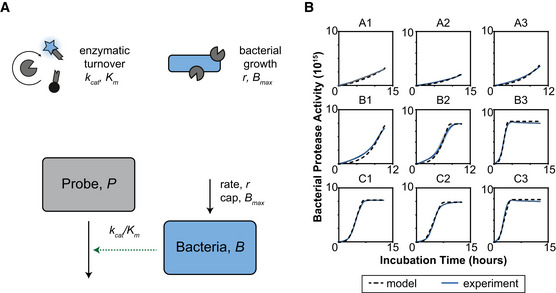
Calculating bacterial growth rate and enzymatic activity in the AMP prodrug system Schematic of the simplified model that only includes bacteria and a substrate probe, which was used to quantify *r* and *k_cat_* from bacterial cleavage assays.Bacterial cleavage assays (blue line) where we are measuring the increase in fluorescence from the cleavage of the fluor‐quencher substrate probe over time. Bacteria and fluor‐quencher probe are incubated together for 12 h. Then, we fit a simplified model to the results, which is plotted as the dashed line. Each panel is labeled with the environmental condition as described in Table EV3 and correlating with Fig 4.

**Figure EV3:**
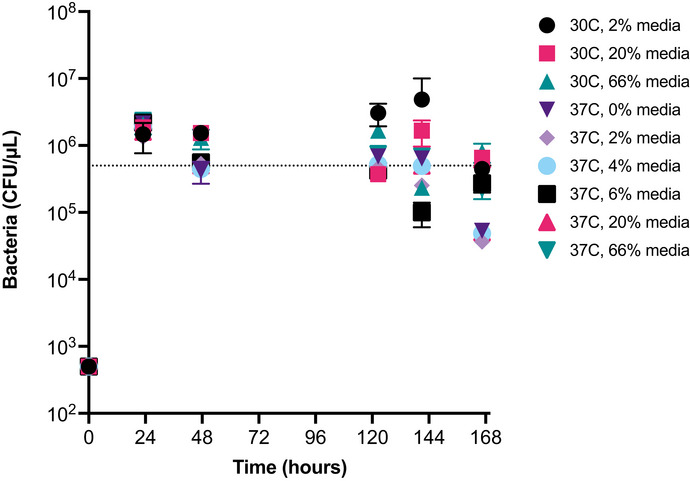
Bacterial carrying capacity measurements for all conditions in the AMP prodrug system Extended kinetic survey of bacterial concentrations at various media conditions to determine effect of temperature and media concentration on this strain of DH5a *E. coli*. Each dot represents the mean bacteria concentration (*n* = 3 biological replicates, CFU/µl, y‐axis, log_10_ axis) at a specific temperature and media concentration (see legend) over time (hours, x‐axis). Dashed line represents the *B_max_* concentration used in the model (y = 5 × 10^5^ CFU/µl). Error bars are plotted as standard deviation.

**Figure EV4:**
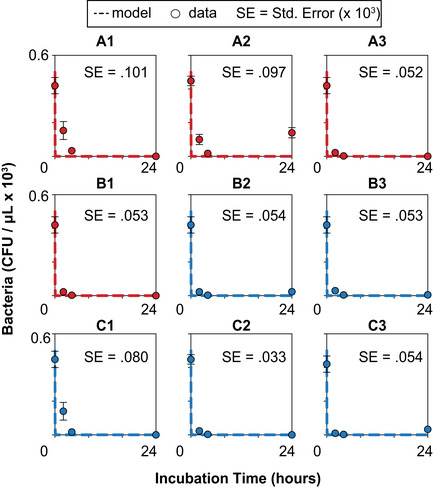
Parent drug control for all conditions in AMP prodrug system Parent drug (R9, polyarginine) controls for conditions testing different media conditions and temperatures in the AMP prodrug studies. Free peptide was incubated with bacteria under the appropriate conditions for 24 h and serial CFU measurements were taken at 0, 2, 4, and 24 h postincubation (red or blue dots; *n* = 3 biological replicates, error bars SEM) and ODE model simulations (red or blue dashed lines) of nine conditions (A1–3, B1–3, C1–3), Standard error (SE) represents the difference between model predictions and experimental observation and is displayed in the top right corner of each panel.

**Figure EV5:**
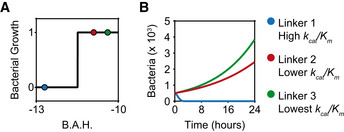
Manipulating the *BAH* by changing the linker sequence on AMP prodrugs Bacteria viability assay post 24 h incubation with drug unlocked by various substrates. Bacterial growth (y‐axis) was equal to 1 if saturating colonies were present after plating (i.e., biofilm), and bacterial growth = 0 if there were no colonies present (blue, green, red dots; *n* = 3). Bacterial growth rates were held constant, and therefore *BAH* values (x‐axis) were calculated using the different *k_cat_* values associated with each linker. Solid black line represents the values predicted by the model as seen in Fig 4.Bacteria growth kinetics (green line, red line, blue line) predicted by the model, simulating 24 h for each of the three linkers. Substrate sequences: Linker 1 = RRSRRV, Linker 2 = RKTR, Linker 3 = ENLYFQG.

We sought to fit our computational model to this experimental bacteria‐prodrug system (AMP prodrug + DH5α *E. coli*) (Fig 4A) and demonstrate that the dimensionless parameter *BAH* can predict which conditions are favorable to prodrug success. Rather than using global parameter fitting method after the final system was tested (i.e., bacteria + prodrugs), we individually measured the values for each of the relevant parameters experimentally, including enzymatic efficiency (e.g., *k_cat_, K_M_*), bacterial growth (e.g., *r*, *B_max_*), and prodrug activity (e.g., *a, b*) in isolated systems (e.g., bacteria alone, enzymes alone, bacteria + activity‐probe, etc.) (Figs EV1, EV2, EV3, Tables EV2 and EV3). This allowed us to more rigorously test the model by predicting bacteria‐prodrug response curves across nine distinct combinations of *k_cat_* and *r* values before performing the physical experiments. We experimentally controlled the nine distinct combinations of *k_cat_* and *r* values by altering the ambient temperature and concentration of broth (conditions labeled A1–3, B1–3, and C1–3; Table EV3). We affected the enzymatic activation rate of the prodrug, *k_cat_,* by changing temperature as described by the Arrhenius equation (Calvert, 1990). Our model anticipated two possible steady‐state outcomes to prodrug treatment; bacteria were predicted to be either susceptible to the prodrug and die or to escape prodrug treatment and proliferate to saturating levels (Fig 3). To experimentally validate this, we incubated bacteria with AMP prodrug under the defined nine conditions and quantified the number of living bacteria longitudinally over the course of a 24‐h treatment window. Quantified bacterial counts taken during treatment closely matched the values predicted by our model (red and blue dots; Figs 4B and C, and EV4). Furthermore, our model predicted that the dimensionless parameter, *BAH*, would separate prodrug success conditions from prodrug failure conditions. We calculated the *BAH* values for each of the nine conditions (Table EV3) and plotted against the final bacteria number (normalized by *B_max_*), which revealed that a critical *BAH* (*BAH_crit_*) clearly predicted the conditions where prodrug treatment was favorable (Fig 4D). By receiver‐operating‐characteristic (ROC) analysis, *BAH_crit_* perfectly predicted the conditions where prodrug treatment succeeded (AUROC = 1.00, *n* = 9) with 100% specificity and sensitivity. By comparison, the unlocked drug control (i.e., free polyarginine) successfully treated bacteria in all nine conditions tested (Fig EV4). We next sought to demonstrate an experimental example of how the *BAH_crit_* could be used to guide successful prodrug treatment. Our model results predicted that changing key system parameters to decrease the *BAH* below the critical threshold will result in successful treatment of bacteria. To demonstrate this, we took three different AMP prodrugs with distinct linker sequences (Table EV1), which served to increase *k_cat_* values for OmpT, thereby decreasing the *BAH* value below *BAH_crit_*. By treating the same population of bacteria with a prodrug that has a slightly faster activation rate, we were able to successfully treat bacteria which previously escaped prodrug treatment (Fig EV5). Collectively, these experiments demonstrate that when *E. coli* are exposed to the AMP prodrug, our model can be used to predict bacterial growth kinetics that closely match experimental observation. Furthermore, the *BAH* is a robust predictor of high‐level outcomes (i.e., success or failure) across the treatment conditions tested.

### Validating the model and predicting prodrug success with TM‐TMP and UTI89 *E. coli*

**Figure 5:**
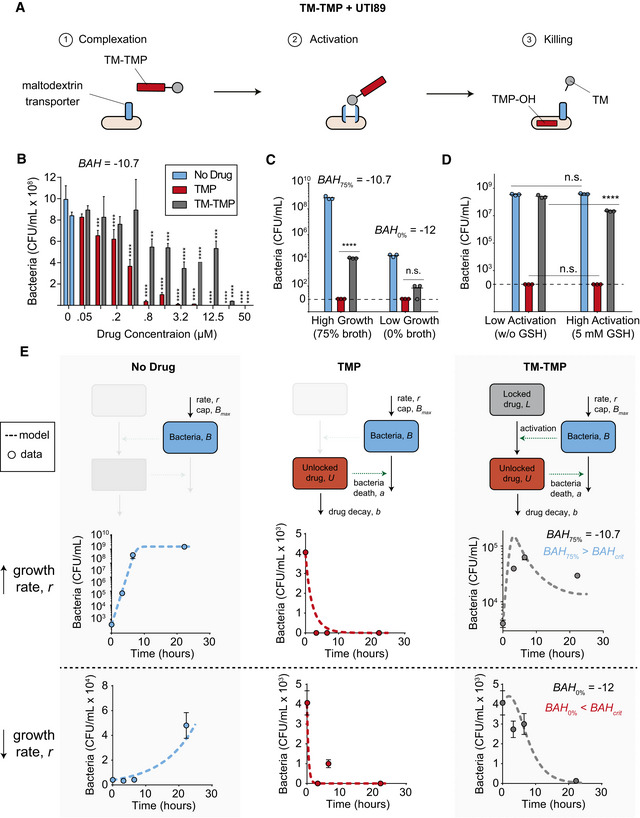
Validating the model and predicting prodrug success with TM‐TMP and UTI89 *E. coli* Schematic of the activation mechanism for the prodrug TM‐TMP with the bacteria UTI89 *E. coli*. The thiomaltose (TM, gray) subcomponent first complexes with maltodextrin transporters (blue) on the surface of bacteria (tan) (step 1). The disulfide self‐immolative linkage connecting TM to trimethoprim (TMP, red) is cleaved by thiols, releasing free TMP‐OH and thus activating the drug (step 2). Free TMP‐OH kills the bacteria (step 3).Measuring the toxicity of free TMP (red) and prodrug TM‐TMP (gray) against UTI89, when compared to no treatment (blue). A range of drug concentrations are incubated with bacteria and the final concentration of living bacteria is measured (*n* = 3 biological replicates; bar height = mean, error bars = standard deviation; one‐way ANOVA + Dunnett’s multiple comparisons test).Measuring the number of surviving bacteria under different growth conditions (i.e., high, 75%, or low, 0%, broth concentration). Bacteria plus no drug (blue), free drug (i.e., TMP, red) and prodrug (i.e., TM‐TMP, gray) are incubated in 0% broth (i.e., PBS) or 75% broth (*n* = 3 biological replicates; bar height = mean, error bars = standard deviation; one‐way ANOVA + Tukey’s multiple comparisons test).Measuring the number of surviving bacteria under different drug activation rates (i.e., the presence or absence of glutathione, GSH). Bacteria plus no drug (blue), free drug (i.e., TMP, red) and prodrug (i.e., TM‐TMP, gray) are incubated with no GSH, or 5 mM GSH, which increases the activation rate of the prodrug.Plotting longitudinal measurements of living bacteria over time under different drug treatment conditions. Each column represents the drug treatment (no drug = blue, TMP = red, and TM‐TMP = gray), as labeled in the title. Each row represents the environmental condition affecting growth rate (top row, low growth rate = 0% broth; bottom row, high growth rate = 75% broth). Each plot shows the concentration of bacteria (CFU/ml) over time. Circles with error bars (standard deviation) are experimental measurements (*n* = 3 biological replicates) and dashed lines are predicted by the computational model. Data information: All conditions in (c) and (d) are compared using one‐way ANOVA with multiple comparisons test. All comparisons are made in reference to the bacteria only control (blue bars) **P < 0.01, ***P < 0.001, and ****P < 0.0001.

**Figure EV6:**
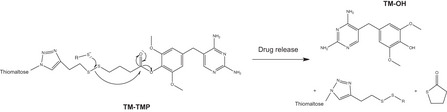
Proposed cleavage of TM‐TMP in the presence of exogenous thiols Schematic adapted from Wang *et al Bioconjug Chem*, 2018. TM‐TMP is inactive (prodrug) when conjugated, but TMP‐OH (active drug) is released upon cleavage by thiols.

**Figure EV7:**
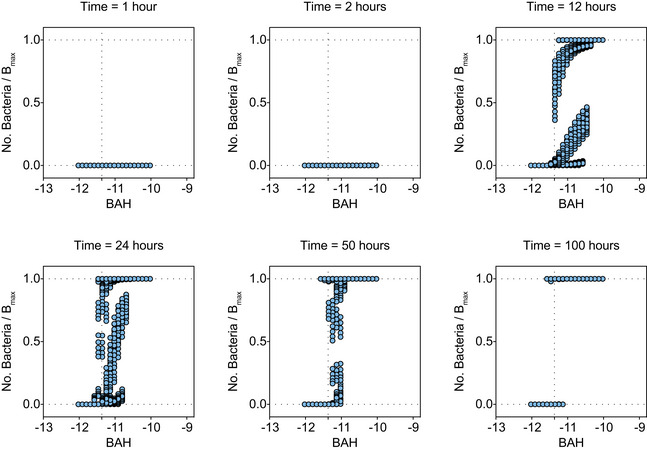
Predicting the *BAH* value for the model TM‐TMP system Computational simulation results of the TM‐TMP system. Each panel represents simulation results from > 2,500 iterations, where *k_cat_*, *K_M_*, *r*, and *B_max_* were varied (blue dots). Each panel represents the results after a certain amount of simulated time (Title: time = x hours). For each simulation (blue dot), a *BAH* (x‐axis) was calculated using *k_cat_* and *r*, and an outcome was plotted as the number of bacteria divided by *B_max_* (y‐axis). The horizontal dashed lines at y = 0 and y = 1 represent the upper and lower limits, and the vertical dashed line represents the estimated *BAH_crit_*.

**Figure EV8:**
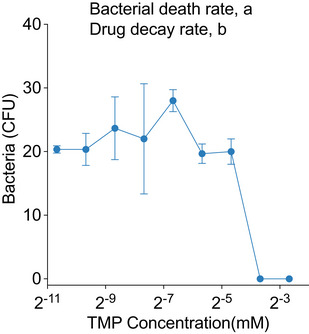
Measuring kinetic parameters for the TM‐TMP Prodrug model Drug dosing experiment plotting the quantity of bacteria surviving after treatment (plotted as CFU, y‐axis) versus the concentration of the parent drug (i.e., TMP; x‐axis). Experiment was taken at short time interval (*t* < 10 min) so we could calculate the number of bacteria and drug copies consumed in each killing reaction. This calculation helps us to estimate the parameters (1) bacterial death rate, *a*, and (2) drug decay rate, *b* for the TM‐TMP prodrug system (*n* = 3 biological replicates).

We next sought to validate our model with an orthogonal bacteria‐prodrug pair, for which we used the strain UTI89 *E. coli*, which has been used in mouse models of urinary tract infections (Mysorekar & Hultgren, 2006; Hung *et al*, 2009), in combination with a thiomaltose (TM)‐conjugated prodrug of the common antibiotic trimethoprim (TMP) (Forsch *et al*, 2004; Ho & Juurlink, 2011), known as TM‐TMP (Wang *et al*, 2018). Conjugating thiomaltose to trimethoprim has been shown to increase the water solubility of TMP by 100‐fold, while being stable to serum enzymes and maintaining activity against urinary tract infections in mice (Wang *et al*, 2018). In this formulation, thiomaltose serves as a targeting ligand by complexing the prodrug with maltodextrin transporters, which are exclusively expressed by bacteria, relative to mammalian cells (Fig 5A, step 1) (Wang *et al*, 2018). Then, thiomaltose is conjugated to TMP via a self‐immolative disulfide linker that releases TMP‐OH, which is as active as TMP, upon disulfide cleavage by free thiols (Fig 5A, step 2; Fig EV6), resulting in the killing of bacteria (Fig 5A, step 3). When comparing the relative bacterial toxicity of TMP (parent drug) and TM‐TMP (prodrug) under one set of environmental conditions (i.e., 37°C, 75% broth), we found that the parent drug was significantly more efficient at killing UTI89 bacteria (Fig 5B; minimum inhibitory concentration (MIC), MIC_TMP_ = 3.2 μM versus MIC_TM‐TMP_ = 50 μM; *n* = 3), which closely matched results from a separate study (Wang *et al*, 2018). We hypothesized that the prodrug was less effective because the BAH value in this experiment was above the critical threshold, indicating conditions favorable to prodrug escape. To test this, we calculated the BAH for this experiment (Fig 5B, *BAH* = −10.7) and found that it was indeed higher than the critical threshold (Fig EV7, *BAH*_crit_ = −11.3), suggesting that prodrug would succeed if the bacterial growth rate was decreased by at least an order of magnitude. To verify this experimentally, we examined the same conditions (e.g., TMP versus TM‐TMP) at one drug concentration (10 μM), but decreased the broth concentration to significantly reduce the bacterial growth rate, *r*, (i.e., approximately 1.23 orders of magnitude; *r_75%_* = 1.7 s^−1^ versus *r_0%_* = 0.1 s^−1^) which decreased the *BAH* value. We observed that by decreasing the *BAH* value below *BAH_crit_* (i.e., *BAH_75%_* = −10.8 versus *BAH_0%_* = −12), the efficacy of the prodrug was significantly increased and the parent drug (TMP) and the prodrug (TM‐TMP) performed more similarly (Fig 5C). To verify this with a second experiment, we also decreased the *BAH* via changing the activation rate of the prodrug, *k_cat_*, by spiking in glutathione (GSH), which rapidly hydrolyzes TM‐TMP (Wang *et al*, 2018). We found that by adding in GSH (5 mM GSH condition), the number of bacteria killed by TM‐TMP (prodrug) was significantly increased, whereas the bacterial counts did not significantly change in either the TMP (parent drug) condition or negative control (no drug) (Fig 5D). These results demonstrated that using the *BAH* as a guiding parameter enabled us to predict the conditions most conducive to prodrug success. Next, we sought to determine whether the computational model associated with the *BAH* (Fig 1) closely matched the kinetics of bacterial growth with this new bacteria‐prodrug pair. To validate the model, we started by testing the bacteria population only (negative control) and then built up to the full model by adding in the unlocked drug and the locked drug populations in a stepwise manner (i.e., (i) no drug, (ii) TMP = unlocked drug, and (iii) TM‐TMP = locked drug) (Fig 5E, top row). For each version of the model (i.e., each column), we tested both a high bacterial growth rate, *r*, (Fig 5E, middle row) and a low bacterial growth rate (Fig 5E, bottom row), which we controlled by changing the broth concentration. First, by measuring the kinetics of the bacteria population alone, we were able to measure key system parameters (e.g., bacterial growth rate, *r*, and bacterial carrying capacity, *B_max_*), which resulted in a close match between experimental and computational results (Fig 5E, left column). Just as with our earlier experiments, we measured (i) the number of bacteria and (ii) the number of drug molecules consumed in each killing reaction to calculate the parameters *a* and *b*, respectively (Fig EV8). Using these parameter values, we used the computational model to predict bacteria population kinetics at high and low growth rates when dosed with TMP (parent drug), which closely matched our experimental results (Fig 5E, middle column). Finally, both the model and the *BAH* value correctly predicted whether the TM‐TMP (prodrug) would successfully treat bacteria (i.e., population decays over time) or whether the bacteria would escape treatment (i.e., population grows over time; Fig 5E, right column). These results confirm that our model matches experimental kinetics and that the *BAH* parameter can be used to predict conditions that favor prodrug success.

## Discussion

The advantages of prodrugs (e.g., increased solubility, pathogen targeting, etc.) are becoming more widely recognized in contemporary drug design (Rautio *et al*, 2018b). While there have been many studies on bacterial resistance strategies that affect traditional antibiotics (i.e., parent drug), comparatively little attention has been given to studying success/failure conditions specific to prodrugs. To study success and failure conditions in prodrugs, we developed a mathematical model of the competition between bacterial growth and prodrug activation rates. We found that our general model fit the experimental observations from both *in vitro* prodrug‐bacteria systems well, while only modifying parameter values between systems. However, future work may improve the model by testing systems with distinct structures such as multistep activation mechanisms, multiple bacterial phenotypes, or dynamic parameter values. While this work held parameters *a* and *b* constant within each system (Tables EV2 and EV4), the model predictions could be further improved by measuring these constants under all environmental conditions. Furthermore, subsequent iterations may also incorporate different models for drug killing (e.g., E_max_) (Holford, 2017) or bacterial growth (de Jong *et al*, 2017).

**Figure EV9:**
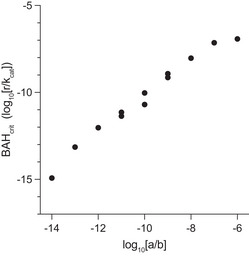
Simulating the dependence of BAH_crit_ on *a* and *b* Using the computational model, we generated five linearly spaced values for parameter *a* between 5 × 10^−15^ µl/h and 5 × 10^−11^ µl/h and for parameter *b* between 5 × 10^−5^ µl/h and 5 × 10^−1^ µl/h. For all permutations of these values for *a* and *b*, we calculated the BAH_crit_ value by varying all other system parameters (e.g., *r*, *B_max_*, *k_cat_*, *K_M_*) and finding the point at which the steady‐state switches from 0 to *B_max_*. We then plotted the base‐10 log of the ratio between *a* and *b* (x‐axis) against the BAH_crit_ (y‐axis) for each iteration.

From our model, we derived a dimensionless parameter, *BAH*, that predicted the transition between prodrug escape and successful treatment. We found that these prodrugs failed in conditions where bacterial growth outpaced the rate of prodrug activation, as predicted by our computational results. This feedback (i.e., feedback loop; Fig 1B) between bacterial density and drug concentration is similar to the feedback between bacterial density and antibiotics in the inoculum effect (Tan *et al*, 2012), or the feedback between drug‐insensitive cells and drug‐sensitive cells in multidrug adaptive therapies for cancer (West *et al*, 2020). We demonstrated that both environmental (e.g., temperature, available nutrients) and pharmacokinetic (e.g., activation rate *k_cat_*) parameters can be tuned to engineer successful prodrug therapies. These findings may reveal opportunities for improvement in prodrug design; for example, this information could be leveraged to improve the efficacy of existing prodrugs by tuning the rate of prodrug decay (i.e., biological half‐life), which influences the *BAH_crit_* transition value (Fig EV9). Alternatively, the catalytic efficiency of the prodrug substrate could be tuned to increase the probability of success, which has been previously demonstrated by engineering prodrug substrates with higher affinity for the enzymatic target (Jordan *et al*, 1999; Barak *et al*, 2006). Importantly, the *BAH* provides a quantitative target for such design modifications and is specific to the nature of prodrugs.

By comparison, previous studies which focused solely on bacterial resistance to the parent form of antibiotics yielded parameters that may not apply to prodrug forms. For example, the minimum inhibitory concentration (MIC) is commonly used as a parameter for resistance (Brauner *et al*, 2016), yet the MIC of the parent and pro‐forms of the same drug can differ, as seen with both model prodrugs used in this study as well as others from the literature (Wang *et al*, 2018; Evans *et al*, 2019; Yang *et al*, 2021). Using existing MIC classifications, these prodrugs could have been labeled ineffective; yet, our experiments showed that different environmental conditions or, in the case of AMP prodrugs, linker sequences, may result in success. One possible reason for the discrepancy in MIC in our studies is that there are kinetic parameters (e.g., drug activation rate) which do not apply to the parent drug, but are key driving factors in determining the outcome of prodrug treatment. Future iterations of this work and the *BAH* may result in a standardized quantitative design criteria that is specific to prodrugs. This is supported by the fact that even with nonprodrugs, metrics such as the single‐cell MIC (Artemova *et al*, 2015) and others based on bacterial temporal dynamics (Meredith *et al*, 2015) have been developed for predicting treatment outcomes where MIC fails.

Dimensionless parameters like the *BAH* are commonly used in engineering to create metrics that are consistent across unit‐systems (e.g., metric versus imperial) and scales (i.e., the relative size of the variables). In the AMP prodrug system, we found that the transition between prodrug escape and successful treatment occurred sharply at one value (i.e., *BAH*_crit_ = −11.3). By comparison, in the TM‐TMP system this transition occurred across a range of values (i.e., −11.5 < *BAH*_crit_ < −11.1), which mirrors the example of pipe flow where the transition from laminar to turbulent flow occurs across a range of Reynolds numbers (i.e., 2,300 < Re < 4,000). Interestingly, both the *BAH*_crit_ values (−11.3 versus −11.5 to −11.1) and the ratios of *a* to *b* (0.5 × 10^−11^ versus 3 × 10^−11^; Tables EV2 and EV4) were similar between systems, which is consistent with our simulations predicting the dependence of the *BAH*_crit_ value on the ratio *a* to *b* (Fig EV9). Furthermore, dimensionless parameters measured in model systems can be used to make predictions about scaled‐up versions of the same system (i.e., similitude). Analogously, future work may show that the *BAH* could be used to predict which prodrugs are most likely to succeed in clinical settings based on smaller scale preliminary studies.

In clinical settings, prodrug failure could potentially be caused by environmental perturbations (e.g., temperature, pH, etc.) as demonstrated in this work, or by genetic mutations that affect pathogen growth rates (Jin *et al*, 2012) or enzymatic activity (Kramer *et al*, 2000, 2001). We predict that mutations affecting the prodrug‐activating enzyme (i.e., *k_cat_*) are the more likely cause of prodrug failure because these mutations are localized to one protein, rather than a cascade of events as in the case of growth rate (*r*) (Jin *et al*, 2012). Based on protein expression numbers alone (Thomassin *et al*, 2012) the range of effective *k_cat_* values is at least one to two orders of magnitude higher on average than the range of potential growth rate values (Allen & Waclaw, 2019; Weissman *et al*, 2021), which means mutations affecting enzyme activity can have a larger impact on the *BAH* value. Furthermore, there are multiple examples of clinical prodrugs with known bacterial resistance mechanisms linked to enzyme mutations. For example, the nitroimidazole class of antibiotics (e.g., metronidazole, dimetridazole, tinidazole, etc.), which is used to treat anaerobic bacteria (e.g., *Enterococcus* species, *Clostridium* species, *Helicobacter pylori*, etc.) represent prodrugs that are activated by bacterial reductases (Edwards, 1993). Genetic studies have revealed that bacterial resistance to nitroimidazole antibiotics is caused by either partial or complete reduction in expression of genes (e.g., rdxA, frxA, etc.) encoding the reductases that activate the prodrug (Jenks *et al*, 1999; Marais *et al*, 2003; Leiros *et al*, 2004). As another example, the major cause of resistance to nitrofuran prodrugs are mutations to *nfsA* and *nfsB*, which are the enzymes responsible for activating the nitrofuran compound (Le & Rakonjac, 2021).

Here, we quantitatively studied the driving parameters that predict the transition between prodrug escape and successful treatment. We envision that this body of work will improve the process of prodrug development by providing a quantitative metric for predicting success, ultimately helping to reduce the burden of antibiotic failure.

## Materials and Methods

### Protease cleavage assays

All protease cleavage assays were performed with a BioTek Cytation 5 Imaging Plate Reader, taking fluorescent measurements at 485/528 nm (excitation/emission) for read‐outs measuring peptide substrates terminated with FITC (Fluorescein isothiocyanate). Kinetic measurements were taken every minute over the course of 60–120 min at 37°C. Tobacco etch virus protease (TEVp), along with its substrate and buffer was obtained from Anaspec, Inc. (Fremont, CA). Activity RFU measurements were normalized to time 0 measurement, and as such represent fold change in signal. Outer Membrane Protease T (i.e., OmpT, Protease 7) was purchased from Lifespan Biosciences (Seattle, WA). OmpT fluorescent peptide substrate was custom ordered from Genscript (Piscataway, NJ).

### Bacterial culture and cytotoxicity measurement

DH5α *Escherichia coli* were a gift from Todd Sulchek's BioMEMS lab at Georgia Tech. *E. coli* were cultured in LB broth (Lennox) at 37°C and plated on LB agar (Lennox) plates. LB broth was purchased from Millipore Sigma (Burlington, MA) and LB agar was purchased from Invitrogen (Carlsbad, CA). AMP and locked AMP were custom ordered from Genscript (Piscataway, NJ). See Table EV1 for more information. Bacteria were grown to a concentration of 10^9^ CFU/ml before being used for experiments. Concentration was estimated by measuring the OD_600_ of the bacterial suspension, and assuming an OD_600_ of 1.000 corresponds to a concentration of 8 × 10^8^ CFU/ml. Bacterial cell viability was measured by making eight 10‐fold serial dilutions, and plating three 10‐µl spots on an LB agar plate. Plates were incubated overnight at 37°C, and CFUs were counted. Untreated bacteria CFU counts served as control for 0% cytotoxicity, and bacteria + IPA (or 0 countable CFUs) served as control for 100% cytotoxicity.

### Computational model

The ODE modelling and solutions were performed in MATLAB 2020b. Code can be found in supplementary information.

### Statistical analysis

Statistical analysis was performed using statistical packages included in GraphPad Prism 6. To assess the significance of an increase in signal due to protease cleavage, we used a two‐way ANOVA (without repeated measures) followed by Sidak's multiple comparisons test. A one‐way ANOVA followed by Dunnett's multiple comparisons test was used to compare experimental means to cells only control bacterial viability assays. Two‐way ANOVA followed by Sidak's multiple comparisons test used to compare experimental means to control for bacterial cytotoxicity at multiple starting concentrations.

### Supplementary analysis

To determine which steady‐state solution is the stable steady‐state for our model, we perform a linear stability analysis on the system of differential equations.

First, we rewrite the model in dimensionless variables and parameters:

$$\dot{X}/k_{\mathrm{cat}}=\pi_{1}X\left(1‐X\right)‐\pi_{2}XZ$$

$$\dot{Y}/k_{\mathrm{cat}}=‐\pi_{3}X\frac{Y}{1+Y}$$

$$\dot{Z}/k_{\mathrm{cat}}=\pi_{3}X\frac{Y}{1+Y}‐\pi_{4}XZ$$

Where the dimensionless variables are defined as:

$$X=\frac{B}{B_{\max}},\;\;\;\;Y=\frac{L}{K_{M}},\;\;\;\;Z=\frac{U}{K_{M}}$$

And the dimensionless parameters are defined as:

$$\pi_{1}=\frac{r}{k_{\mathrm{cat}}},\;\;\;\;\;\;\pi_{2}=\frac{aK_{M}}{k_{\mathrm{cat}}},\;\;\;\;\;\;\pi_{3}=\frac{B_{\max}}{K_{M}},\;\;\;\;\;\;\pi_{4}=\frac{bB_{\max}}{k_{\mathrm{cat}}}$$

Steady‐state Solution 1: For the steady‐state solution where *B* = 0, the dimensionless variable solutions that follow are:

$$X=0,\;\;\;\;\;Y=\frac{1}{\frac{\pi_{2}\pi_{3}}{\pi_{1}\pi_{4}}‐1},\;\;\;\;\;\;\;\;Z=\frac{\pi_{1}}{\pi_{2}}$$

The resulting Jacobian, *J*, and eigenvalues, *λ*, are:

$$J=\left[\begin{array}{ccc} 0 & 0 & 0\\ ‐\pi_{1}\pi_{4}/\pi_{2} & 0 & 0\\ 0 & 0 & 0 \end{array}\right],\;\;\;\;\;\lambda =0,0,0$$

Steady‐state Solution 2: For the steady‐state solution where *B* = *B_max_*, the dimensionless variable solutions that follow are:

X=1,Y=0,Z=0

The resulting Jacobian, *J*, and eigenvalues, *λ*, are:

$$J=\left[\begin{array}{ccc} ‐\pi_{1} & 0 & 0\\ 0 & ‐\pi_{3} & 0\\ 0 & \pi_{3} & ‐\pi_{4} \end{array}\right],\;\;\;\;\;\;\;\lambda =‐\pi_{1},‐\pi_{3},‐\pi_{4}$$

Since the eigenvalues for steady‐state solution 2 (i.e., *B* = *B_max_*) are all negative, whereas the eigenvalues for steady‐state solution 1 (*B* = 0) are all equal to 0, we can conclude that steady‐state solution 2 is stable and steady‐state solution 1 is unstable.

## Author contributions

GAK, NM, and BAH contributed ideas. KGA, BAH, MT, MS, and YX designed experiments and interpreted results. BAH, MT, YX, MS, JJR, and XW carried out experiments. GAK and BAH wrote the manuscript. BAH, MT, YX, MS, NM, and GAK edited the manuscript.

## Supporting information



Expanded View Figures PDF

Table EV1

Table EV2

Table EV3

Table EV4

## Data Availability

The data supporting the findings of this study are available within the paper and EV files. The code is available within the EV files and at https://github.com/brandon‐holt/bacterial‐advantage‐heuristic.
