# Supplementary material for: Dimensionless parameter predicts bacterial prodrug success
Source: Mol Syst Biol. 2022 Jan 10;18(1):e10495. doi: 10.15252/msb.202110495 (PMC8744131; doi:10.15252/msb.202110495)
Supplement: Supplementary file 1 — Expanded View Figures PDF [file MSB-18-e10495-s002.pdf]

Expanded View Figures

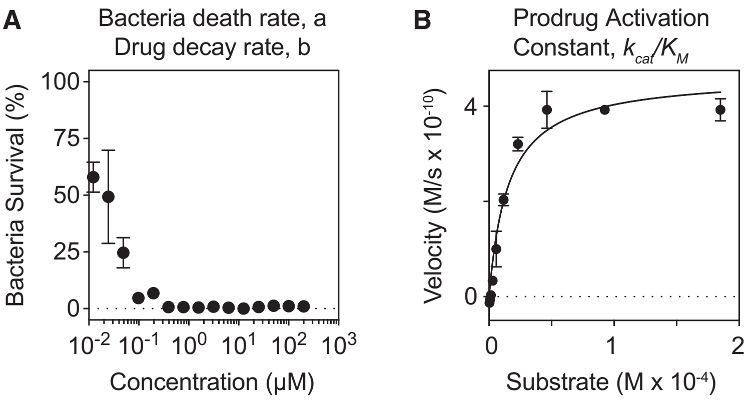

Figure EV1. Measuring kinetic parameters for the AMP Prodrug model.

A Drug dosing experiment plotting percent of bacteria surviving (CFU count divided by number of CFUs in no drug control) versus the concentration of the parent drug (i.e., free AMP, polyarginine). Experiment was taken at a short time interval ($t < 10$ min) so we could calculate the number of bacteria and drug copies consumed in each killing reaction. This calculation helps us to estimate the parameters (1) bacterial death rate, *a*, and (2) drug decay rate, *b* for the AMP prodrug system.

B Michaelis–Menten experiment with recombinant OmpT and the AMP prodrug linker substrate. At various concentrations of substrate, we measured the initial velocity (substrates cleaved over time) of the reaction (black dots). Then, we fit the Michaelis–Menten equation to these results (black line) to calculate the k_{cat} and K_M values for this enzyme–substrate pair.

Data information: Error bars are plotted as standard deviation, $n = 3$ biological replicates.

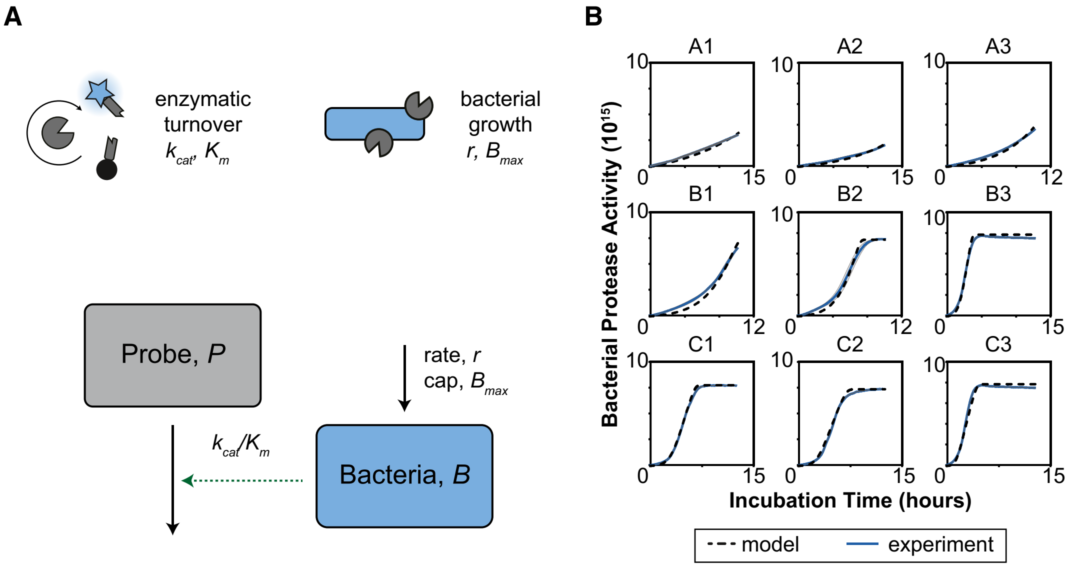

Figure EV2. Calculating bacterial growth rate and enzymatic activity in the AMP prodrug system.

A Schematic of the simplified model that only includes bacteria and a substrate probe, which was used to quantify *r* and k_{cat} from bacterial cleavage assays.

B Bacterial cleavage assays (blue line) where we are measuring the increase in fluorescence from the cleavage of the fluor-quencher substrate probe over time. Bacteria and fluor-quencher probe are incubated together for 12 h. Then, we fit a simplified model to the results, which is plotted as the dashed line. Each panel is labeled with the environmental condition as described in Table EV3 and correlating with Fig 4.

© 2022 The Authors
14 of 13 Molecular Systems Biology 18: e10495 | 2022 © 2022 The Authors

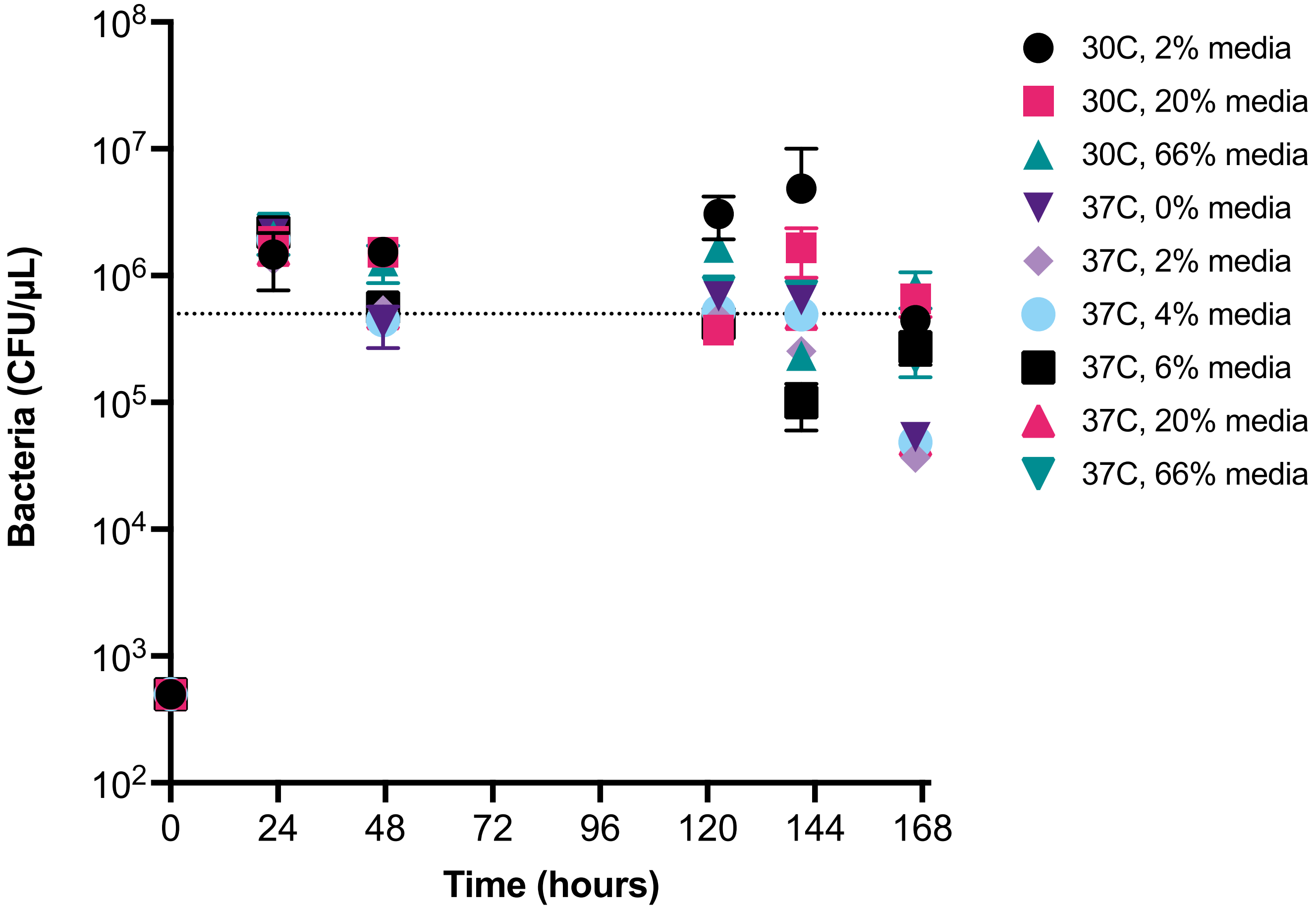

Figure EV3. Bacterial carrying capacity measurements for all conditions in the AMP prodrug system.

Extended kinetic survey of bacterial concentrations at various media conditions to determine effect of temperature and media concentration on this strain of DH5a *E. coli*. Each dot represents the mean bacteria concentration ($n = 3$ biological replicates, CFU/µl, y-axis, $\log_{10}$ axis) at a specific temperature and media concentration (see legend) over time (hours, x-axis). Dashed line represents the B_{max} concentration used in the model ($y = 5 \times 10^5$ CFU/µl). Error bars are plotted as standard deviation.

© 2022 The Authors

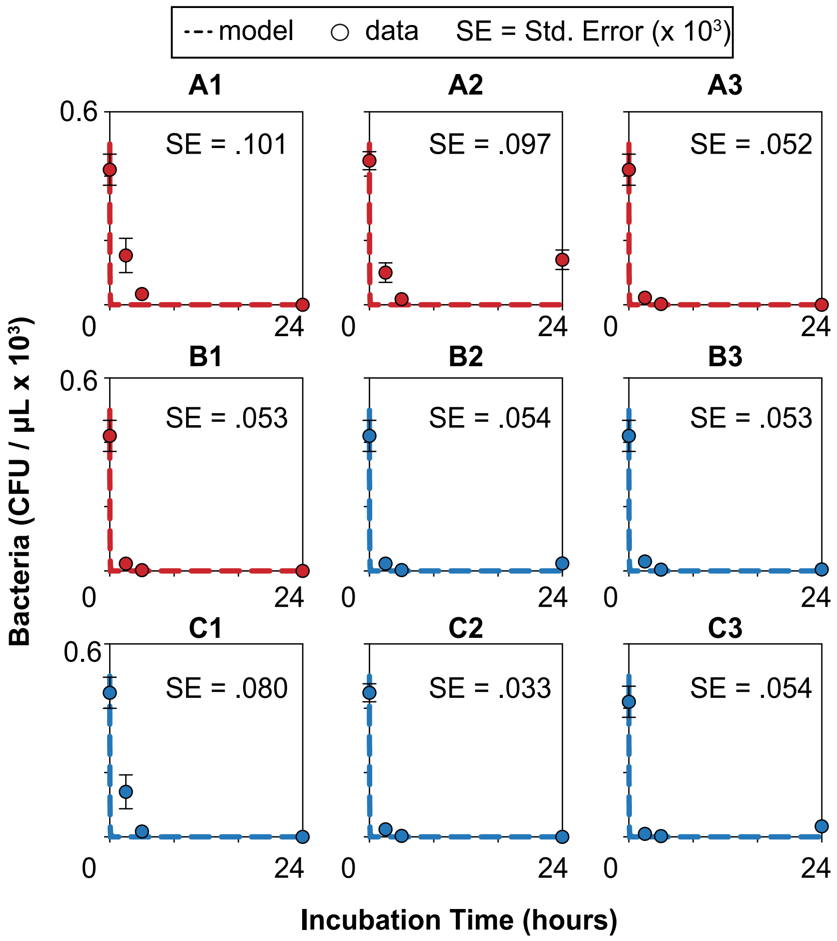

Figure EV4. Parent drug control for all conditions in AMP prodrug system.

Parent drug (R9, polyarginine) controls for conditions testing different media conditions and temperatures in the AMP prodrug studies. Free peptide was incubated with bacteria under the appropriate conditions for 24 h and serial CFU measurements were taken at 0, 2, 4, and 24 h postincubation (red or blue dots; $n = 3$ biological replicates, error bars SEM) and ODE model simulations (red or blue dashed lines) of nine conditions (A1–3, B1–3, C1–3), Standard error (SE) represents the difference between model predictions and experimental observation and is displayed in the top right corner of each panel.

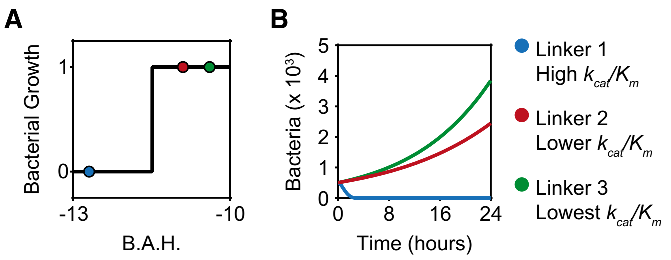

Figure EV5. Manipulating the *BAH* by changing the linker sequence on AMP prodrugs.

A Bacteria viability assay post 24 h incubation with drug unlocked by various substrates. Bacterial growth (y-axis) was equal to 1 if saturating colonies were present after plating (i.e., biofilm), and bacterial growth = 0 if there were no colonies present (blue, green, red dots; $n = 3$). Bacterial growth rates were held constant, and therefore *BAH* values (x-axis) were calculated using the different k_{cat} values associated with each linker. Solid black line represents the values predicted by the model as seen in Fig 4.

B Bacteria growth kinetics (green line, red line, blue line) predicted by the model, simulating 24 h for each of the three linkers. Substrate sequences: Linker 1 = RRSRRV, Linker 2 = RKTR, Linker 3 = ENLYFQG.

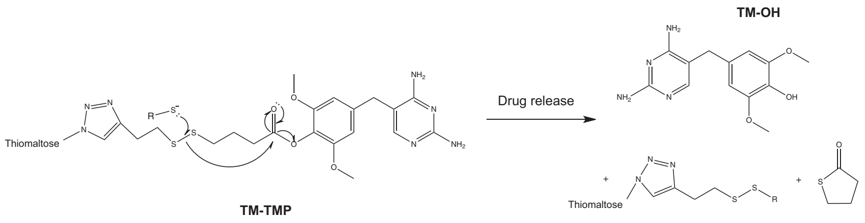

Figure EV6. Proposed cleavage of TM-TMP in the presence of exogenous thiols.

Schematic adapted from Wang *et al Bioconjug Chem*, 2018. TM-TMP is inactive (prodrug) when conjugated, but TMP-OH (active drug) is released upon cleavage by thiols.

© 2022 The Authors

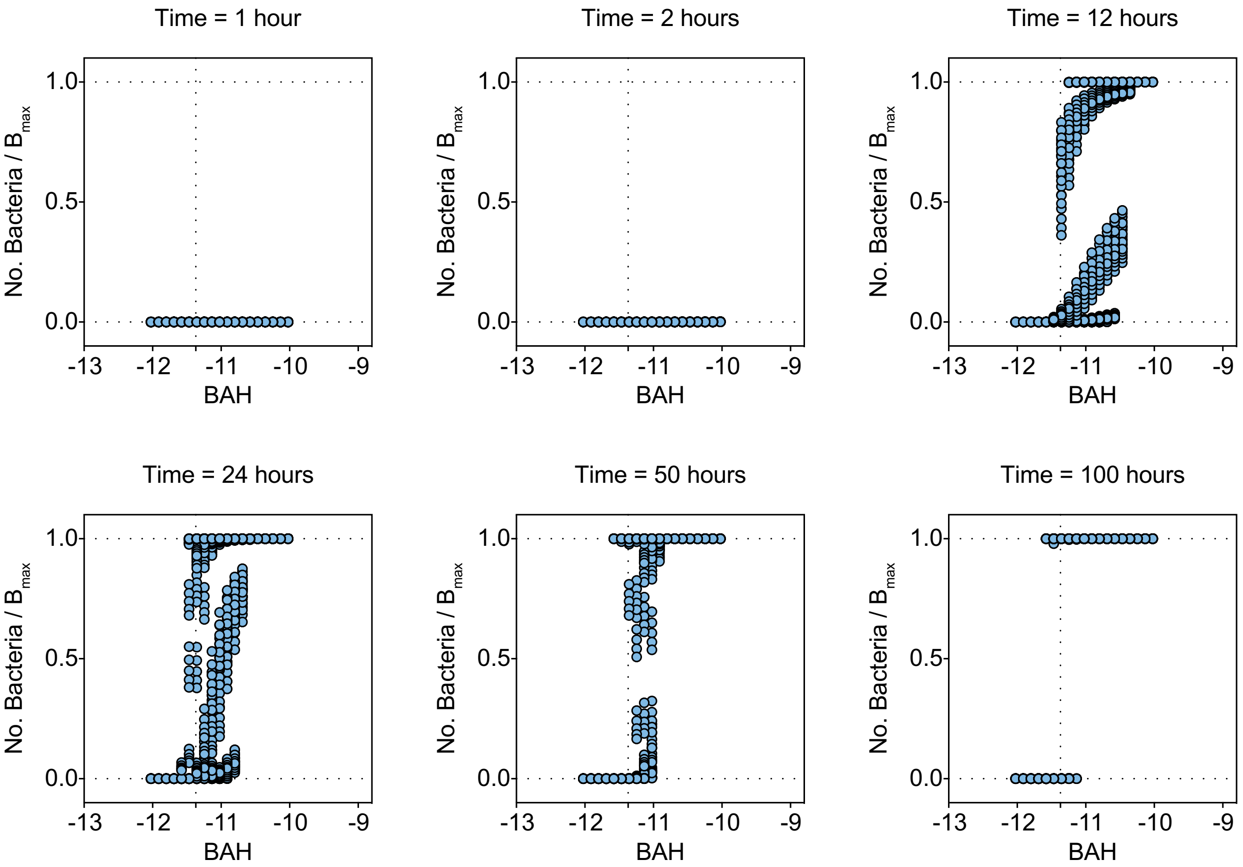

Figure EV7. Predicting the *BAH* value for the model TM-TMP system.

Computational simulation results of the TM-TMP system. Each panel represents simulation results from > 2,500 iterations, where k_{cat}, K_M, r, and B_{max} were varied (blue dots). Each panel represents the results after a certain amount of simulated time (Title: time = x hours). For each simulation (blue dot), a *BAH* (x-axis) was calculated using k_{cat} and r, and an outcome was plotted as the number of bacteria divided by B_{max} (y-axis). The horizontal dashed lines at y = 0 and y = 1 represent the upper and lower limits, and the vertical dashed line represents the estimated BAH_{crit}.

© 2022 The Authors

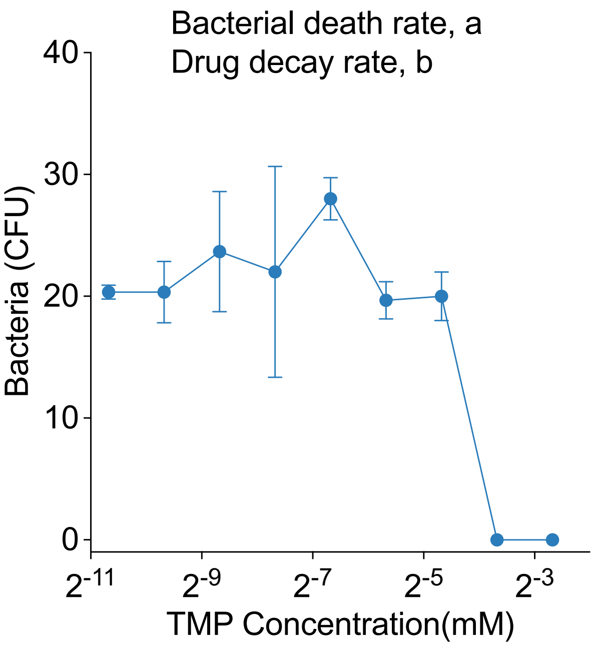

Figure EV8. Measuring kinetic parameters for the TM-TMP Prodrug model.

Drug dosing experiment plotting the quantity of bacteria surviving after treatment (plotted as CFU, y-axis) versus the concentration of the parent drug (i.e., TMP; x-axis). Experiment was taken at short time interval ($t < 10$ min) so we could calculate the number of bacteria and drug copies consumed in each killing reaction. This calculation helps us to estimate the parameters (1) bacterial death rate, *a*, and (2) drug decay rate, *b* for the TM-TMP prodrug system ($n = 3$ biological replicates).

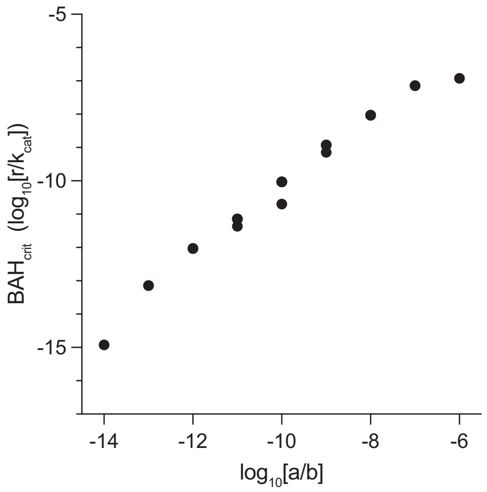

Figure EV9. Simulating the dependence of BAH_{crit} on *a* and *b*.

Using the computational model, we generated five linearly spaced values for parameter *a* between 5×10^{-15} µl/h and 5×10^{-11} µl/h and for parameter *b* between 5×10^{-5} µl/h and 5×10^{-1} µl/h. For all permutations of these values for *a* and *b*, we calculated the BAH_{crit} value by varying all other system parameters (e.g., *r*, B_{max}, k_{cat}, K_M) and finding the point at which the steady-state switches from 0 to B_{max}. We then plotted the base-10 log of the ratio between *a* and *b* (x-axis) against the BAH_{crit} (y-axis) for each iteration.

© 2022 The Authors
