# Supplementary material for: Dimensionless parameter predicts bacterial prodrug success
Source: Mol Syst Biol. 2022 Jan 10;18(1):e10495. doi: 10.15252/msb.202110495 (PMC8744131; doi:10.15252/msb.202110495)
Supplement: Supplementary file 2 — Table EV1 [file MSB-18-e10495-s001.docx]

**TABLE EV1**

| **Name** | **Peptide Sequence** |
| --- | --- |
| Locked AMP (linker 1) | EEEEEEEEEEEEERRSRRVRRRRRRRRR |
| Locked AMP (linker 2) | EEEEEEEEEEERKTRRRRRRRRR |
| Locked AMP (linker 3) | EEEEEEEEENLYFQGRRRRRRRRR |
| Locked AMP Probe | [DABCYL]-EEEEEEEEEEEEERRSRRVRRRRRRRRR[Lys(5-FAM)] |
| OmpT Probe | [DABCYL]-RRSRRV-[Lys(5-FAM)] |

**Table EV1. Peptide sequences.** Name of peptide sample (first column from left) referenced in manuscript and amino acid sequence (second column from left) of corresponding sample. All uppercase letters are single-letter amino acid codes. All motifs in square brackets represent either a quencher compound (e.g., [DABCYL]) or a fluorophore compound (e.g., [Lys(5-FAM)]). Locked AMPs are named according to the legend in **Fig. EV5**. Linker 1 is the default locked AMP peptide sequence used if not specified. Locked AMP probe is the peptide used in **Fig. 3d**. OmpT Probe is used in all other cleavage assays to measure OmpT activity.

[DABCYL] = 4-((4-(dimethylamino)phenyl)azo)benzoic acid

[Lys(5-FAM)] = Lysine, 5 – Carboxyfluorescein
