# Supplementary material for: Dimensionless parameter predicts bacterial prodrug success
Source: Mol Syst Biol. 2022 Jan 10;18(1):e10495. doi: 10.15252/msb.202110495 (PMC8744131; doi:10.15252/msb.202110495)
Supplement: Supplementary file 4 — Table EV3 [file MSB-18-e10495-s006.docx]

**TABLE EV3**

| **Condition Label** | **Temperature** | **LB Broth Concentration** | ***r* (h^-1^)** | ***k_cat_* (h^-1^) x 10^10^** | ***BAH*** |
| --- | --- | --- | --- | --- | --- |
| A1 | 37 ˚C | 0% | 0.1 | 25 | -12.39 |
| A2 | 30 ˚C | 2% | 0.14 | 13.6 | -11.98 |
| A3 | 37 ˚C | 2% | 0.3 | 22 | -11.86 |
| B1 | 37 ˚C | 4% | 0.5 | 15 | -11.47 |
| B2 | 37 ˚C | 6% | 0.85 | 13.6 | -11.20 |
| B3 | 37 ˚C | 20% | 2.2 | 1 | -9.65 |
| C1 | 30 ˚C | 20% | 1.4 | 5.2 | -10.56 |
| C2 | 30 ˚C | 66% | 1.6 | 4 | -10.39 |
| C3 | 37 ˚C | 66% | 3 | 6 | -10.30 |

**Table EV3. Additional information for experimental conditions.** Table shows specific experimental values for each condition label (first column from left) shown in **Fig. 4b**. This information includes incubation temperature (second column from left), the concentration of LB broth by volume (third column from left), growth rate, *r* (fourth column from left), enzyme catalytic turnover, *k_cat_* (fifth column from left), and the bacterial advantage heuristic, *BAH* (sixth column from left).
