# Supplementary material for: Dimensionless parameter predicts bacterial prodrug success
Source: Mol Syst Biol. 2022 Jan 10;18(1):e10495. doi: 10.15252/msb.202110495 (PMC8744131; doi:10.15252/msb.202110495)
Supplement: Supplementary file 5 — Table EV4 [file MSB-18-e10495-s005.docx]

**TABLE EV4**

| **Parameter** | **Value** | **Units** | **Expanded Name** |
| --- | --- | --- | --- |
| *r* | 0.1—0.7 | h^-1^ | Bacterial growth rate |
| *k_cat_* | 1e11 | h^-1^ | Enzyme catalytic turnover |
| *K_M_* | 16 | µM | Michaelis constant |
| *B_max_* | 1.5e9 | CFU * mL^-1^ | Max bacteria concentration in tube |
| *a* | 3e-16 | mL * h^-1^ | Bacterial death rate constant |
| *b* | 1e-5 | mL * h^-1^ | Drug decay rate constant |

**Table EV4. Parameters used in UTI89 + TM-TMP prodrug model.** Parameter symbol (first column from left), value or range of values used for each parameter (second column from left), parameter units (third column from left), and expanded name of parameter as used in manuscript text (fourth column from left) are shown in the table.
