# Supplementary material for: Dimensionless parameter predicts bacterial prodrug success
Source: Mol Syst Biol. 2022 Jan 10;18(1):e10495. doi: 10.15252/msb.202110495 (PMC8744131; doi:10.15252/msb.202110495)
Supplement: Supplementary file 6 [file MSB-18-e10495-s004.pdf]

Dimensionless parameter predicts bacterial prodrug success

Brandon Holt, McKenzie Tuttle, Yilin Xu, Melanie Su, Joachim Roise, Xioajian Wang, Niren Murthy, and Gabe Kwong
DOI: 10.15252/msb.202110495

Corresponding author(s): Gabe Kwong (gkwong@gatech.edu)

Review Timeline:

Submission Date:	9th Jun 21
Editorial Decision:	27th Jul 21
Revision Received:	25th Oct 21
Editorial Decision:	9th Dec 21
Revision Received:	15th Dec 21
Accepted:	17th Dec 21

Editor: Maria Polychronidou

Transaction Report:

(Note: With the exception of the correction of typographical or spelling errors that could be a source of ambiguity, letters and reports are not edited. Depending on transfer agreements, referee reports obtained elsewhere may or may not be included in this compilation. Referee reports are anonymous unless the Referee chooses to sign their reports.)

Thank you again for submitting your work to Molecular Systems Biology. I apologize for the delay in getting back to you, which was due to the late arrival of the referee reports. We have now heard back from the three referees who agreed to evaluate your study. Overall, the reviewers acknowledge that the topic and findings are relevant. They raise however a series of concerns, which we would ask you to address in a revision.

I think that the recommendations of the reviewers are rather clear and therefore I do not see the need to repeat the points listed below. All issues raised would need to be satisfactorily addressed. Please contact me in case you would like to discuss in further detail any of the issues raised.

On a more editorial level, we would ask you to address the following points:

REFEREE REPORTS

Reviewer #1:

In this manuscript, Holt et al. describe the observation that under certain conditions bacteria are capable of surviving and proliferating rapidly in the presence of prodrug concentrations that would normally kill them. Based on this observation, the

authors develop a kinetic model to describe the possible sources of resistance, including increased growth rate or decreased protease activity (since proteases are often responsible for activating the prodrug). This model relies on a simple dimensionless parameter called the bacterial advantage heuristic (BAH) which relates the bacterial growth rate to the prodrug activation rate, essentially saying how fast the bacteria need to grow to "outrun" activation of the prodrug. Notably, they build the model by first measuring the relevant parameters in isolation rather than fitting them after the fact, ensuring that the model is physiologically relevant and accurate. They then test the model on two different prodrugs, varying nutrient content and temperature to validate the model under a variety of conditions, showing that experimental results confirm model predictions. Notably, they confirm mechanism of prodrug activation by knocking out OmpT to show it is essential for activation and killing.

Overall, I thought this was an excellent manuscript that was clearly prepared in terms of both text and figures. The phenomenon of prodrug resistance is interesting from a mechanistic biological perspective and is understudied. The quantitative model is intuitive to understand and relies on minimal fitting, ensuring that it is physiologically relevant. I would recommend publication of the manuscript with only minor revisions that could be addressed with limited additional text, likely in the discussion.

Specific Comments:

1) How much of prodrug resistance is likely result from modulating activation rate vs. modulating growth rate? If distinct adaptation mechanisms exist for resistance, what are the types of mutations that might drive these adaptations? How do these mutations compare and contrast to known resistance mechanisms for the parent drug? Is there reason to believe that prodrugs are much less prone to mutation since cells only "see" the activated form for a short window before killing?

2) Thinking about the above, how likely is this form of prodrug resistance to occur in clinically relevant conditions? For example, is this form of resistance due to outpacing of growth a likely outcome in other strains and conditions that don't have the fast growth rate of lab-adapted E. coli? If growth rates are slower (due to nutrient limitation, anaerobic conditions, competition etc.) could this still occur?

Reviewer #2:

In this study, Holt et al. investigate how bacterial activation rates of prodrug antibiotics influence bacterial killing. To this aim, the authors establish a system of ordinary differential equations describing the bacterial growth rate, prodrug activation rate by bacteria, antibacterial killing by the activated/unlocked antibiotic prodrug, and inactivation/decay of the antibiotics. Using their model, the authors define conditions under which the prodrug activation becomes rate limiting and hence hinders antibiotic success. To synthesize these findings, the authors then define the term 'Bacterial Advantage Heuristics, BAH' describing the ratio of bacterial growth rate over prodrug activation rate, which leads to successful prodrug antibiosis when below a certain threshold value. In the experimental part of this study, the authors use two prodrug antibiotics to validate their model with E. coli grown under different culture conditions.

Although rather academic, I found the addressed question and the chosen approach in principle interesting. However, some of the experimental approaches to parametrize and validate the model might need to be improved:

Major Comments.
1) The experimental conditions chosen to validate the model are not ideal and crucial control experiments are missing:

i) Diluted culture media not only affects growth rates, but also maximum bacteria concentration (Bmax) and the duration of the growth phase in batch culture. It is unclear how these two effects were taken into account in the model. I propose to use a cleaner experimental system to assess the effect of growth rate alteration on probiotic activation and antibiotic efficiency using substrate-uptake titration, as previously reported for lactose uptake by E. coli (https://doi.org/10.1038/s41586-020-2505-4).

ii) The authors report that protease activity (kcat) is altered under different growth conditions, but an explanation for this observation is lacking and the rational for employing these specific experimental conditions to modulate kcat is missing. I strongly suggest to use different promotors to tightly control OmpT expression in a OmpT knock out background to systematically assess the impact of kcat on probiotic activation and the antibiotic effect.

iii) The different temperatures and media conditions impact bacterial physiology and as a consequence the bacterial death rate (a) and drug decay rate (b). It remains unclear, how the alteration of these parameters was taken into account for each of the growth conditions.

iv) Control experiments with the unlocked prodrug (at different concentrations) are missing for each of the nine conditions/growths curves shown in Figure 4b.

2) The description of the second experimental example using TM-TMP is incomplete leading to some experimental flaws. Rather than the activation of the prodrug, which results from the reaction with free thiol groups, the rate-limiting step seems to be the

prodrug transport into bacterial cells, which should hence be assessed in the model. However, the authors either add GSH or the unlocked prodrug to the bacterial cultures for validation (Fig. 5d and 5e). This is a clear flaw, as both manipulations lead to the extracellular increase of the unlocked prodrug and hence neither mimic altered intracellular TM-TMP activation, nor the (likely rate limiting) TM-TMP transport into the cell.

Minor Comments.

3) The authors state in the introduction that "Prodrugs represent ~10% of all FDA-approved drugs." Although correct, this statement implies that antibiotic prodrugs are a major drug class. However, this is not the case and most prodrugs are designed to increase oral bioavailability and their activation typically occurs by abundant human enzymes following intestinal absorption. For these drugs, prodrug activation investigated in this study does not seem to be a major issue. I suggest that the authors are more precise in their statement about the role of antibiotic prodrugs in the larger context of FDA-approved (pro)drugs.

4) The authors state: "To apply MM kinetics, we assumed our system constituted a well-mixed solution of freely diffusing substrates (i.e., locked drug) in large excess relative to the number of bacteria-enzymes, which were valid assumptions for our downstream studies since prodrugs were present at concentrations ~10^2 micromolar in an aqueous environment relative to a maximum bacterial concentration of ~10-6 micromolar." It is unclear, why the molecular concentration of bacteria and not of enzymes, which is orders of magnitudes higher, is used for this molecular comparison. Given the high turnover rate leading to the fast accumulation of unlocked prodrug (which is required for the antibiotic effect), MM kinetics seems a wrong assumption to model this parameter over an extended duration of time.

Reviewer #3:

The manuscript by Holt and colleagues investigates escape of bacterial populations from treatment with prodrugs, which become pharmacologically active in the presence of the target pathogen. Using a kinetic model of treatment, they introduce a dimensionless parameter (BAH) that can be used to predict treatment escape and then verify those predictions in two in vitro models, one based on a protease-activated antimicrobial peptide and the other on a modified version of the antibiotic trimethoprim.

This is an interesting manuscript that addresses an important and timely problem. Because prodrug effects can differ from those of the parent drug, it is difficult to predict the overall treatment success using classical measures like MIC (even more difficult than in the typical case, where MIC is already an over-simplified metric). In this case, the complexity arises primarily due to feedback between the prodrug activity and the dynamics of the pathogen population, giving rise to potentially counterintuitive effects. The authors have addressed this problem using a simple model and a nice set of experiments. While the model obviously neglects many features of the full system-as any good model should-it seems to do very well in predicting the primary outcome of interest, and the intuitive insight gained by the BAH parameter is both convenient and powerful. Overall, I think this is a nicely done study, but I have several comments that I think would significantly improve the work (and shouldn't be too difficult to implement).

-- The model is sufficiently simple (a strength, not a weakness!) that I think you should be able to do a linear stability analysis of the B=0 solution that would confirm the numerical results of Figure 2. It might also give you additional insight into the critical value of BAH and the effect of the other system parameters on that critical value (I suspect the critical value does depend on these other parameters, even if that dependence is weak over the studied parameter regime). I think it would be worthwhile to first write the model in dimensionless variables / parameters (r/kcat will be one of them) and then do a linear stability analysis.

-- Related to the comment above: the current model will have L=0 in the steady state since there is no source term for increasing L in equation 1.2, no? Since the locked drug becomes unlocked, and the unlocked drug decays, the drug will eventually disappear and the cells will re-grow (since the population can never hit extinction in this model), right? One option is to develop a stochastic version of the model that accounts for demographic noise and would allow for true extinction. But a much simpler approach is to consider adding a source (constant) term to Equation 1.2 that provides an influx of locked drug. Then you have a well-defined steady state solution that (presumably) bifurcates as r/kcat crosses some threshold. To compare to experiments, one option might be to choose that influx term so that the steady state of L matches the concentration you used in the experiment (or some estimate of its steady state concentration on the experimental timescale). One could then think of that model as valid on the experimental timescale, at least in the regime where the concentration of prodrug reaches some quasi steady state. On longer timescales, of course, the prodrug itself will decay, but then you are forced to study only the transient behavior of the model with numerics, as the steady state will be less interesting and always result in full growth and zero drug. If you add the source term and do the stability analysis, I think you'll find that the critical value of r/kcat depends on some of the other parameters through the (quasi) steady state value of L. My hunch is that the values you choose represent combinations of these parameters that could be predicted by this analysis.

-- The feedback between population density and drug concentration seems to be driving many of the interesting results seen here. The authors may wish to comment on / connect their results to other known cases from the literature where similar

feedback loops (driven by population density) contribute to the way bacteria respond to antibiotics. For example, similar feedback loops play a role in the classical inoculum effect (IE) as well as in adaptive therapies, which attempt to maintain drug sensitive populations as a competitive barrier to the emergence of resistance populations (these adaptive therapies have been studied in cancer, parasitic infections, and antibiotic resistance). These connections may open the work to a broader audience interested in antibiotic resistance and also raise interesting questions for future work (at least in my opinion).

Minor points:
-- It might be nice to mention other studies that indicate how MIC alone is not always a good proxy for treatment efficacy, even for regular (not pro-) drugs. For example, metrics such as single-cell MIC (Jeff Gore lab) or other time dependent measures (L. You lab) have been developed for predicting treatment outcomes in cases where resistance is cooperative. I think the discussion could be broadened to include a bit more on this well-studied topic (and this does not detract from the novelty of the current study).

Authors' Responses to Reviewer Points

Reviewer #1:

Point 1. *"In this manuscript, Holt et al. describe the observation that under certain conditions bacteria are capable of surviving and proliferating rapidly in the presence of prodrug concentrations that would normally kill them. Based on this observation, the authors develop a kinetic model to describe the possible sources of resistance, including increased growth rate or decreased protease activity (since proteases are often responsible for activating the prodrug). This model relies on a simple dimensionless parameter called the bacterial advantage heuristic (BAH) which relates the bacterial growth rate to the prodrug activation rate, essentially saying how fast the bacteria need to grow to "outrun" activation of the prodrug. Notably, they build the model by first measuring the relevant parameters in isolation rather than fitting them after the fact, ensuring that the model is physiologically relevant and accurate. They then test the model on two different prodrugs, varying nutrient content and temperature to validate the model under a variety of conditions, showing that experimental results confirm model predictions. Notably, they confirm mechanism of prodrug activation by knocking out OmpT to show it is essential for activation and killing. Overall, I thought this was an excellent manuscript that was clearly prepared in terms of both text and figures. The phenomenon of prodrug resistance is interesting from a mechanistic biological perspective and is understudied. The quantitative model is intuitive to understand and relies on minimal fitting, ensuring that it is physiologically relevant. I would recommend publication of the manuscript with only minor revisions that could be addressed with limited additional text, likely in the discussion…"*

Response 1. We appreciate that the reviewer has performed a thorough evaluation of the manuscript and recommends publication. We have updated the discussion to include text that addresses the reviewer's comments. We outline these changes and respond to the reviewer's specific questions in the responses below.

Point 2. *"…How much of prodrug resistance is likely result from modulating activation rate vs. modulating growth rate?..."*

Response 2. In physiological conditions, we believe it is more likely that prodrug resistance would be caused by mutations affecting the expression or efficiency of the prodrug-activating enzymes compared to mutations affecting growth rate. We hypothesize that mutations affecting one specific enzyme are less likely to causes secondary issues with other aspects of bacterial survival than a mutation affecting such a high-level function such as growth rate, which involves a cascade of events (Jin, Cagliero et al. 2012). However, this would not be the case if the prodrug-activating enzyme is known to be required for specific bacterial survival functions.

Additionally, based on measurements of bacterial growth-rates in our work (**Table EV2**) and by others (Allen and Waclaw 2019, Weissman, Hou et al. 2021), values for *r* appear to span at most one order of magnitude. By contrast, the minimum possible range k_{cat} can span is determined by the range of possible copy numbers of the prodrug-activating enzyme. For example, up to 10^2's of copies of OmpT are expressed (Thomassin, Brannon et al. 2012) and there is no lower limit on the expression of OmpT since OmpT-knockout bacteria are capable of proliferation (**Fig. 3b**). For OmpT, the range of possible k_{cat} values should be at least two orders of magnitude but could be even higher depending on the range of possible mutations that can increase enzymatic efficiency, which is difficult to predict. In this example, because the range of possible k_{cat}values is at least an order of magnitude larger than the range of *r* values, mutations affecting enzyme activity can

have a larger impact on the *BAH* value. Therefore, we hypothesize that in this example it would be more probable that a mutation affecting bacterial resistance to prodrugs would result from changes to k_{cat} compared to *r*. We have added text addressing our hypotheses regarding how much of prodrug resistance is likely to result from modulating activation rate vs. growth rate:

"We predict that mutations affecting the prodrug-activating enzyme (i.e., kcat) are the more likely cause of prodrug failure because these mutations are localized to one protein, rather than a cascade of events as in the case of growth rate (r). Based on protein expression numbers alone the range of effective kcat values is at least one to two orders of magnitude higher on average than the range of potential growth rate values, which means mutations affecting enzyme activity can have a larger impact on the BAH value."

Point 3. *"…If distinct adaptation mechanisms exist for resistance, what are the types of mutations that might drive these adaptations? How do these mutations compare and contrast to known resistance mechanisms for the parent drug?..."*

Response 3. Genetic mutations that affect 1) E. coli growth rates (Jin, Cagliero et al. 2012) or 2) the expression of proteases responsible for prodrug activation (Kramer, Zandwijken et al. 2000, Kramer, Vandeputte-Rutten et al. 2001) could drive prodrug escape in the drugs studied in our work. For traditional antibiotics (i.e., non-prodrugs) in general, this is comparable to inherited resistance, which arises from genetic mutations that alter key bacterial processes including metabolism, enzymatic activity, and drug efflux pumps (McKeegan, Borges-Walmsley et al. 2002). For the parent drugs used in this study, there are no established resistance mechanisms for the AMP drug (i.e., polyarginine) because it is not a clinically-administered antibiotic; however, there are known resistance mechanisms to TMP (i.e., trimethoprim). The resistance mechanisms to TMP include: (1) the permeability barrier and/or efflux pumps, (2) naturally insensitive target enzymes, (3) regulational changes in the target enzymes, (4) mutational or recombinational changes in the target enzymes, and (5) acquired resistance by drug-resistant target enzymes (Eliopoulos and Huovinen 2001). To clarify, the enzymes mentioned here are not referring to prodrug-activating enzymes; they are referring to dihydrofolate reductase (DHFR), for which TMP acts as an inhibitor and is a key enzyme in bacterial DNA synthesis. Resistance mechanisms affecting the permeability barrier would be most similar to the types of mutations that would also affect the prodrug form (TM-TMP) studied in our work, since they would result in changes to the effective k_{cat} value and the resulting *BAH*. The rest of the mechanisms, which involve the target enzyme DHFR, should affect TMP and TM-TMP equally since the interaction with DHFR is downstream of the prodrug activation step. We have added text addressing the types of mutations that might drive these adaptations in a clinical setting to the discussion section:

"In clinical settings, prodrug failure could potentially be caused by environmental perturbations (e.g., temperature, pH, etc.) as demonstrated in this work, or by genetic mutations that affect pathogen growth rates or enzymatic activity."

Point 4. *"…Is there reason to believe that prodrugs are much less prone to mutation since cells only "see" the activated form for a short window before killing?..."*

Response 4. It is unlikely that bacteria exposed to prodrugs would be less prone to mutation or that there would be a significant difference between the rate of mutations in bacteria exposed to parent drugs versus prodrugs. From a populational perspective, the only factor that determines whether or not mutations would be considered useful is if they contribute to the survival of the bacteria under the selective pressure of the drug. From the bacteria's perspective, the fact that a

copy of an active drug may have recently been converted from an inactive form before physically interacting with the bacteria should not matter. Whether or not the bacteria survives the physical interaction is determined by the properties of the drug and the fitness of the bacteria. However, the area of mutations and resistance in bacteria under exposure to prodrug therapies is an understudied area, so there is limited data to justify such a hypothesis in either direction.

Point 5. *"…Thinking about the above, how likely is this form of prodrug resistance to occur in clinically relevant conditions? For example, is this form of resistance due to outpacing of growth a likely outcome in other strains and conditions that don't have the fast growth rate of lab-adapted E. coli? If growth rates are slower (due to nutrient limitation, anaerobic conditions, competition etc.) could this still occur?"*

Response 5. Theoretically, the potential for this form of prodrug resistance to occur should almost always exist. Even in cases where there is a physiological constraint on the maximum possible growth rate, *r*, the prodrug-activating enzyme can be downregulated or can experience a mutation that decreases efficiency (i.e., $k_{cat} \rightarrow 0$). The only constraint on the lower bound of k_{cat} would be the minimum levels of expression or activity required to complete functions required for survival (e.g., DNA synthesis, metabolic processes, etc.), assuming this mutation does not coexist with a separate mutation that replaces any relevant survival functions.

As an example, the nitroimidazole class of antibiotics (e.g., metronidazole, dimetridazole, tinidazole, etc.), which is used to treat anaerobic bacteria (e.g., *Enterococcus* species, *Clostridium* species, *Helicobacter pylori*, etc.) represent prodrugs that are activated by bacterial reductases (Edwards 1993). Genetic studies have revealed that bacterial resistance to nitroimidazole antibiotics is caused by either partial or complete reduction in expression of genes (e.g., rdxA, frxA, etc.) encoding the reductases that activate the prodrug (Jenks, Ferrero et al. 1999, Marais, Bilardi et al. 2003, Leiros, Kozielski-Stuhrmann et al. 2004). As another example, the major cause of resistance to nitrofuran prodrugs are mutations to *nfsA* and *nfsB*, which are the enzymes responsible for activating the nitrofuran compound (Le and Rakonjac 2021).

This suggests an interesting therapeutic strategy, where prodrug-activating proteins or enzymes should be chosen that are also required for important bacterial survival functions. This way, bacteria with mutations that decrease k_{cat} and promote prodrug escape would be less likely to survive and proliferate due to a missing survival function. This strategy could be further improved by combining with a bacteriostatic antibiotic to limit growth rate.

We have added text addressing the potential for this form of prodrug failure to occur in clinically relevant conditions to the discussion section:

"In clinical settings, prodrug failure could potentially be caused by…For example, the nitroimidazole class of antibiotics (e.g., metronidazole, dimetridazole, tinidazole, etc.), which is used to treat anaerobic bacteria (e.g., Enterococcus species, Clostridium species, Helicobacter pylori, etc.) represent prodrugs that are activated by bacterial reductases. Genetic studies have revealed that bacterial resistance to nitroimidazole antibiotics is caused by either partial or complete reduction in expression of genes (e.g., rdxA, frxA, etc.) encoding the reductases that activate the prodrug. As another example, the major cause of resistance to nitrofuran prodrugs are mutations to nfsA and nfsB, which are the enzymes responsible for activating the nitrofuran compound."

Reviewer #2:

Point 1. *"In this study, Holt et al. investigate how bacterial activation rates of prodrug antibiotics influence bacterial killing. To this aim, the authors establish a system of ordinary differential equations describing the bacterial growth rate, prodrug activation rate by bacteria, antibacterial killing by the activated/unlocked antibiotic prodrug, and inactivation/decay of the antibiotics. Using their model, the authors define conditions under which the prodrug activation becomes rate limiting and hence hinders antibiotic success. To synthesize these findings, the authors then define the term 'Bacterial Advantage Heuristics, BAH' describing the ratio of bacterial growth rate over prodrug activation rate, which leads to successful prodrug antibiosis when below a certain threshold value. In the experimental part of this study, the authors use two prodrug antibiotics to validate their model with E. coli grown under different culture conditions. Although rather academic, I found the addressed question and the chosen approach in principle interesting. However, some of the experimental approaches to parametrize and validate the model might need to be improved…"*

Response 1. We thank the reviewer for performing a thorough review of the manuscript. We have improved the experimental approaches and controls to validate the model and we have added additional text to the discussion; we outline these changes below.

Point 2. *"…The experimental conditions chosen to validate the model are not ideal and crucial control experiments are missing. Diluted culture media not only affects growth rates, but also maximum bacteria concentration (Bmax) and the duration of the growth phase in batch culture. It is unclear how these two effects were taken into account in the model. I propose to use a cleaner experimental system to assess the effect of growth rate alteration on probiotic activation and antibiotic efficiency using substrate-uptake titration, as previously reported for lactose uptake by E. coli (https://doi.org/10.1038/s41586-020-2505-4)..."*

Response 2. To determine the degree to which diluted culture media and temperature affected maximum bacteria concentrations (B_{max}), we performed new experiments measuring viable bacteria concentrations at time scales (i.e., >24 hours) beyond those tested in our previous studies. While these conditions produce variable growth rates before the system has reached steady state (<24 hours, **Fig. EV2**), the new results show that all conditions tested eventually reach the B_{max} value used for the model (5×10^5 CFU/µL, dashed line). We have updated the manuscript to include these results in **Fig. EV3**.

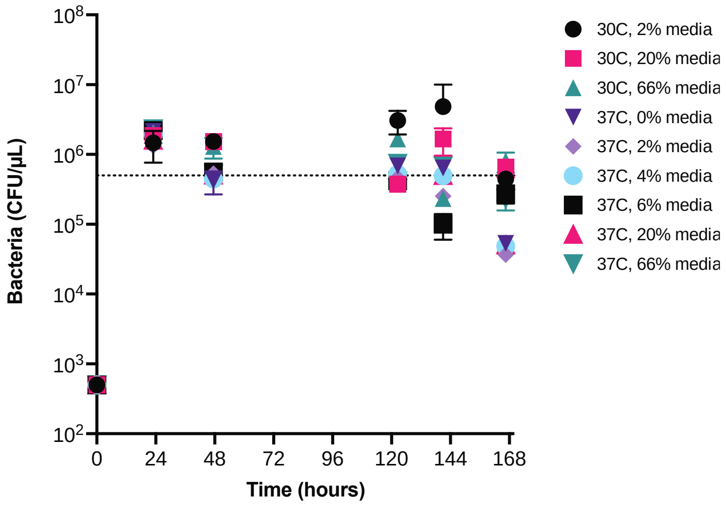

Additionally, we include experiments in our supplemental materials to account for the differences in the growth phase in batch culture. The duration of the growth phase is a function of the inverse of the bacterial growth rate, *r*, which we previously measured for all nine conditions (**Table EV3**).

Further, we agree with the reviewer that changing temperature and media concentrations can potentially change many different variables at once. In fact, this was the exact purpose of this experiment: to determine the extent to which the dimensionless parameter, *BAH*, could predict the conditions that result in prodrug success while many variables are changing. The fact that the *BAH* was able to make these predictions using only two parameters amidst larger system perturbations demonstrates the advantage of using dimensionless parameters. The substrate-uptake titration method would be very interesting to implement in future studies to explore drug-specific mechanisms of prodrug failure/success.

Point 3. *"…The authors report that protease activity (kcat) is altered under different growth conditions, but an explanation for this observation is lacking and the rational for employing these specific experimental conditions to modulate kcat is missing. I strongly suggest to use different promotors to tightly control OmpT expression in a OmpT knock out background to systematically assess the impact of kcat on probiotic activation and the antibiotic effect…"*

Response 3. To clarify, of the growth conditions tested in **Fig. 4**, the primary experimental condition used to modify protease activity was changing incubation temperature. The effect of temperature on enzyme activity can be mathematically described by the Arrhenius equation, which served as a starting point for choosing our experimental conditions:

$$k_{cat} = Ae^{\frac{-E_a}{RT}}$$

Where *A* is the pre-exponential factor, E_a is the activation energy for the reaction, *R* is the universal gas constant, and *T* is temperature.

Secondarily, we observed that different media concentrations also resulted in perturbations in enzyme activity. This is confirmed by our parameter-fitting experiments (**Fig. EV2**, **Table EV3**), which show that under the nine conditions with known growth-rates, changing media concentrations resulted in different k_{cat} values.

We agree that using promoters to tightly control OmpT expression would be a good experiment to assess the impact of k_{cat} on probiotic activation, but this is outside of the scope of our study. Here, rather than explore the effect of each parameter individually, the goal of the study was to determine whether the *BAH*, which is a function of only two parameters, can be used to predict prodrug success when multiple parameters are changing. Such experiments would be more suitable for future studies aiming to elucidate drug-specific resistance mechanisms. To clarify in the manuscript that the primary experimental mechanism for changing k_{cat} was changing temperature, we added the following text to the results section:

"We affected the enzymatic activation rate of the prodrug, kcat, by changing temperature as described by the Arrhenius equation."

Point 4. *"…The different temperatures and media conditions impact bacterial physiology and as a consequence the bacterial death rate (a) and drug decay rate (b). It remains unclear, how the alteration of these parameters was taken into account for each of the growth conditions…"*

Response 4. The reviewer makes an interesting point, which motivated us to perform additional analysis on the model to determine the effect of changing the bacterial death rate, *a*, and the drug decay rate, *b* on the critical *BAH* transition value (**Fig. 4d**). As a reminder, the BAH_{crit} value is the *BAH* value at which the system transitions between prodrug success ($BAH < BAH_{crit}$) and prodrug failure ($BAH > BAH_{crit}$). Using our model, we varied *a* and *b* over four orders of magnitude, and calculated the BAH_{crit} value (y-axis) for each unique ratio of *a* and *b*, which is another dimensionless parameter of this system (x-axis). We observe that there is a linear relationship between these two dimensionless parameters (**Fig. EV9**).

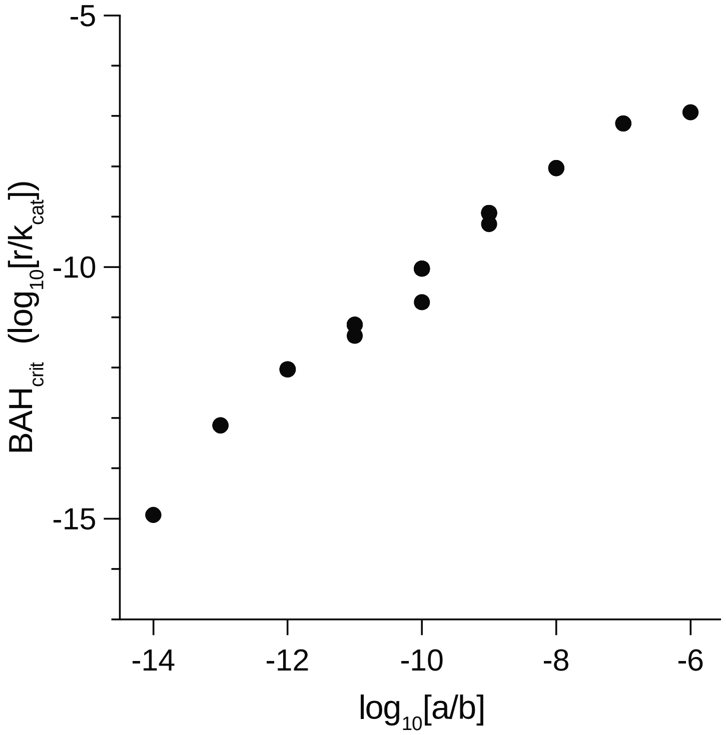

Interestingly, the ratio of *a* to *b* are the primary drivers of the BAH_{crit} value, since previous simulations varying other parameters do not alter the BAH_{crit} value (**Fig. 2c—h**). It is also possible

that the initial conditions affect the BAH_{crit} value, but this has not yet been investigated. In these studies, we measured *a* and *b* independently for both the AMP prodrug system (**Fig. EV1, Table EV2**) and the TM-TMP system (**Fig. EV8, Table EV4**), but we held these values constant within each system. We believe this is a reasonable assumption given that (1) the ratio of these two parameters must change to perturb the steady-state behavior of the system (i.e., *a* and *b* need to change in opposite directions) and (2) the transition BAH_{crit} value predicted by the model closely matched that observed in our experiments (**Fig. 4d, Fig. 5e**). We agree that different temperatures and media conditions may alter these parameters, and to address this we have added text to our discussion section acknowledging this:

*"While this work held parameters a and b constant within each system (**Table EV2, EV4**), the model predictions could be further improved by measuring these constants under all environmental conditions."*

Point 5. *"…Control experiments with the unlocked prodrug (at different concentrations) are missing for each of the nine conditions/growths curves shown in Figure 4b…"*

Response 5. We agree these important control experiments were missing, so we have performed this study and added the data to **Fig. EV4**. The results show that under all nine conditions tested, the free AMP (i.e., parent drug) was capable of treating the bacteria, even in conditions where the prodrug failed to do so (blue traces).

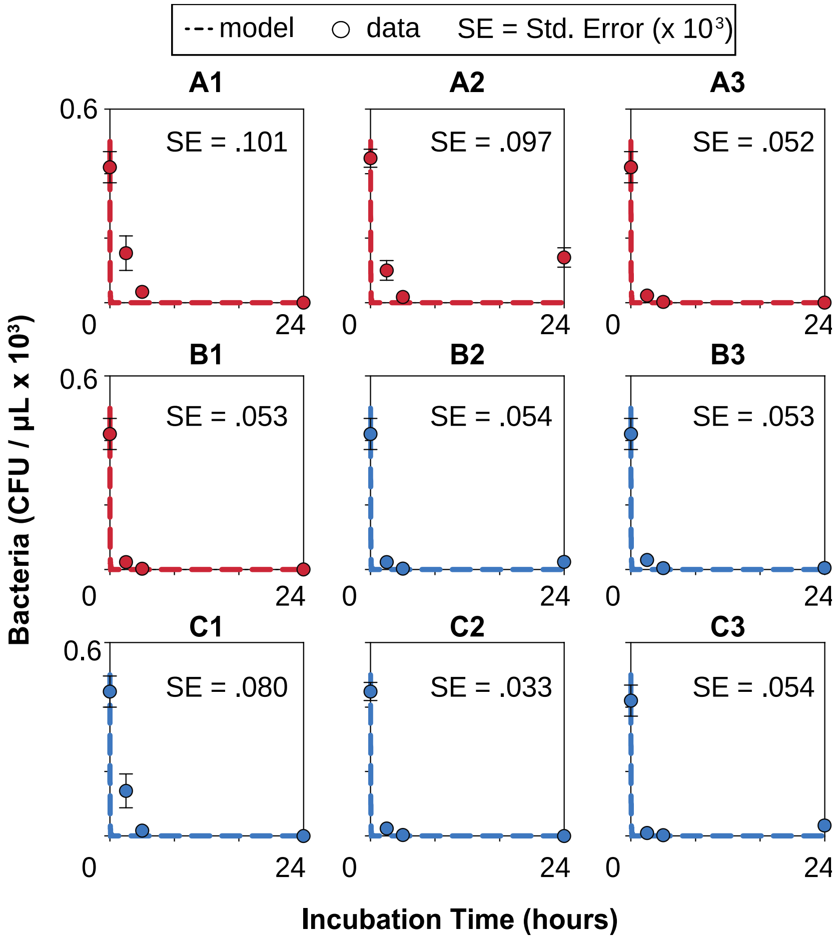

Point 6. *“…The description of the second experimental example using TM-TMP is incomplete leading to some experimental flaws. Rather than the activation of the prodrug, which results from the reaction with free thiol groups, the rate-limiting step seems to be the prodrug transport into bacterial cells, which should hence be assessed in the model. However, the authors either add GSH or the unlocked prodrug to the bacterial cultures for validation (Fig. 5d and 5e). This is a clear flaw, as both manipulations lead to the extracellular increase of the unlocked prodrug and hence neither mimic altered intracellular TM-TMP activation, nor the (likely rate limiting) TM-TMP transport into the cell…”*

Response 6. To clarify, the purpose of adding unlocked prodrug (**Fig. 5e**; TMP) was to serve as a control group for the prodrug condition (TM-TMP). With this control, we showed that changing growth rate impacts the result of the prodrug treatment (TM-TMP), but not the unlocked prodrug treatment (TMP). This supports our hypothesis that an antibiotic failure mechanism specific to prodrugs occurs when the bacterial growth rate outpaces the prodrug activation rate. Further, we showed that the *BAH* value successfully predicted which condition would lead to prodrug success and which would lead to prodrug failure.

We considered adding the transport of TM-TMP (prodrug) into the cells as an additional step in our model. However, we decided it would be more powerful to use one generalized model that applies to both systems. To enable this, we use an effective k_{cat} value, which encompasses all reaction steps involved in activating the prodrug. For example, in the case of TM-TMP, the effective k_{cat} represents both (1) the rate at which the prodrug enters the cell and (2) the drug activation rate. Therefore, by adding GSH (**Fig. 5d**) we increased the rate of the second component of the process modeled by the effective k_{cat} (i.e., the drug activation rate).

Point 7. “…The authors state in the introduction that "Prodrugs represent ~10% of all FDA-approved drugs." Although correct, this statement implies that antibiotic prodrugs are a major drug class. However, this is not the case and most prodrugs are designed to increase oral bioavailability and their activation typically occurs by abundant human enzymes following intestinal absorption. For these drugs, prodrug activation investigated in this study does not seem to be a major issue. I suggest that the authors are more precise in their statement about the role of antibiotic prodrugs in the larger context of FDA-approved (pro)drugs…”

Response 7. We agree that we should be more precise in this statement and we have revised the text to reflect this. We include an excerpt of the revised text here:

“Prodrugs, which represent ~10% of all FDA-approved drugs in the last decade (Rautio, Meanwell et al. 2018), are a promising solution because they present multiple strategies for reviving existing or previously discarded antibiotics (Jubeh, Breijyeh et al. 2020); these strategies include increasing bioavailability and solubility, reducing off-target effects, or targeting bacteria-specific enzymes… In this work we focus specifically on antibiotic prodrugs, a subset of prodrugs that includes compounds such as ganciclovir (Al-Badr and Ajarim 2018) and isoniazid (Metcalfe, Macdonald et al. 2008), which are administered as biologically inactive forms and are enzymatically activated into their parent form by the pathogen.”

Point 8. *“…The authors state: "To apply MM kinetics, we assumed our system constituted a well-mixed solution of freely diffusing substrates (i.e., locked drug) in large excess relative to the number of bacteria- enzymes, which were valid assumptions for our downstream studies since prodrugs were present at concentrations ~10^2 micromolar in an aqueous environment relative to a maximum bacterial concentration of ~10-6 micromolar." It is unclear, why the molecular*

concentration of bacteria and not of enzymes, which is orders of magnitudes higher, is used for this molecular comparison. Given the high turnover rate leading to the fast accumulation of unlocked prodrug (which is required for the antibiotic effect), MM kinetics seems a wrong assumption to model this parameter over an extended duration of time."

Response 8. The reason we report the molecular concentration of bacteria, and not enzymes, is because we model the bacteria as the effective enzymatic unit. We carry this assumption throughout all equations (**Equation 1.2, 1.3**) and measurements of k_{cat} (**Fig. EV1b**).

Additionally, even if we consider the number of enzyme proteins directly, the number of prodrugs would still be in excess relative to the number of OmpT proteins. The number of OmpT proteins would be ~10^2 times higher than the number of bacteria according to mRNA expression profiles (Thomassin, Brannon et al. 2012), so the number of prodrug substrates would be 6 orders of magnitude higher than the number of enzyme proteins (10^2 µM vs. 10^{-4} µM), justifying the use of Michaelis-Menten kinetics.

While there are high turnover rates in certain conditions tested in this paper ($k_{cat} > 10^{11}\ h^{-1}$), this does not always lead to fast depletion of the prodrug (i.e., substrate). For example, in the most extreme case where k_{cat} = 25 x 10^{10} hr^{-1} and growth rate, r = 3 hr^{-1}, the model predicts the prodrug (i.e., locked drug) will be depleted in ~1.1 hrs.

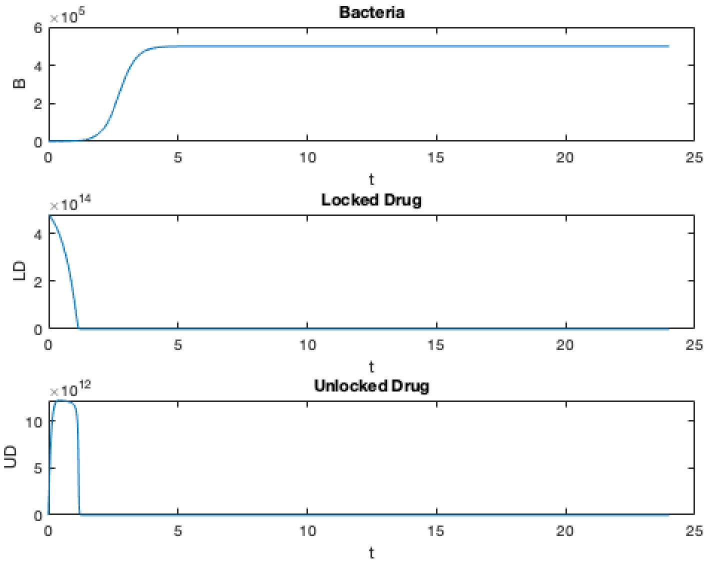

By comparison, if the growth rate is decreased to 0.1, the locked drug (LD) is not depleted before the bacteria population reaches extinction. The LD population only decreases by ~30%.

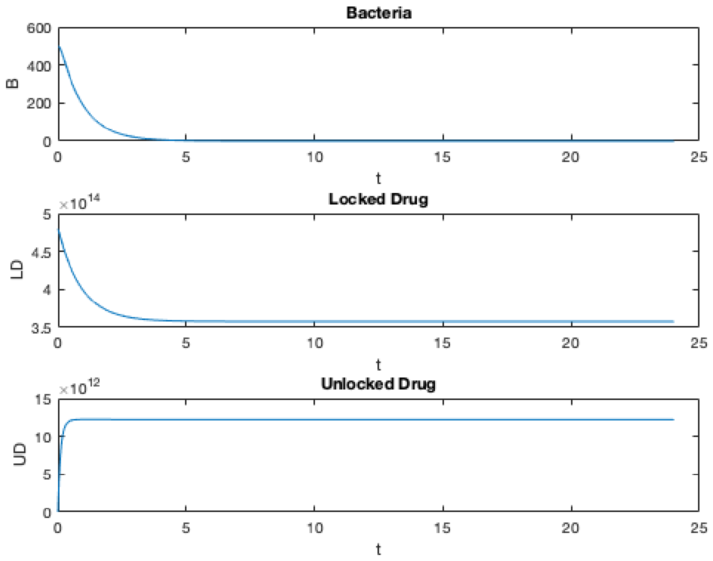

In our work, the result of interest is whether the bacteria survive (i.e., prodrug failure) or die (i.e., prodrug success). In cases where the locked drug is depleted, it does not matter that the prodrug is no longer in excess because the bacteria will survive the treatment regardless of the form of the equation for drug activation (i.e., this term goes to 0). In cases where the locked drug is not rapidly depleted, unlocked drug still accumulates to concentrations sufficient to achieve the antibiotic effect. Therefore, the use of Michaelis-Menten is justified, and in extreme cases where the locked drug is rapidly depleted, this does not affect the end-result of the model.

Reviewer #3:

Point 1. *"The manuscript by Holt and colleagues investigates escape of bacterial populations from treatment with prodrugs, which become pharmacologically active in the presence of the target pathogen. Using a kinetic model of treatment, they introduce a dimensionless parameter (BAH) that can be used to predict treatment escape and then verify those predictions in two in vitro models, one based on a protease- activated antimicrobial peptide and the other on a modified version of the antibiotic trimethoprim. This is an interesting manuscript that addresses an important and timely problem. Because prodrug effects can differ from those of the parent drug, it is difficult to predict the overall treatment success using classical measures like MIC (even more difficult than in the typical case, where MIC is already an over-simplified metric). In this case, the complexity arises primarily due to feedback between the prodrug activity and the dynamics of the pathogen population, giving rise to potentially counterintuitive effects. The authors have addressed this problem using a simple model and a nice set of experiments. While the model obviously neglects many features of the full system-as any good model should-it seems to do very well in predicting the primary outcome of interest, and the intuitive insight gained by the BAH parameter is both convenient and powerful. Overall, I think this is a nicely done study, but I have several comments that I think would significantly improve the work (and shouldn't be too difficult to implement)…"*

Response 1. We thank the reviewer for providing helpful feedback on the computational components of this work and for completing a thorough evaluation of the manuscript. We have performed the linear stability analysis and related experiments to address the reviewer's points. We have also updated the discussion to mention related works as recommended by the reviewer. We outline these changes to the manuscript below.

Point 2. "…The model is sufficiently simple (a strength, not a weakness!) that I think you should be able to do a linear stability analysis of the B=0 solution that would confirm the numerical results of Figure 2. It might also give you additional insight into the critical value of BAH and the effect of the other system parameters on that critical value (I suspect the critical value does depend on these other parameters, even if that dependence is weak over the studied parameter regime). I think it would be worthwhile to first write the model in dimensionless variables / parameters (r/kcat will be one of them) and then do a linear stability analysis…"

Response 2. We have performed a linear stability analysis of the model at both possible steady-state solutions, which include bacterial death (B = 0) and bacterial saturation (B = B_{max}). We have added this analysis to the supplemental information (**Supplementary Analysis**), and we outline the results here:

First, we rewrite the model in dimensionless variables and parameters:

$$\dot{X}/_{k_{cat}} = \pi_1 X(1-X) - \pi_2 XZ$$

$$\dot{Y}/_{k_{cat}} = -\pi_3 X \frac{Y}{1+Y}$$

$$\dot{Z}/_{k_{cat}} = \pi_3 X \frac{Y}{1+Y} - \pi_4 XZ$$

Where the dimensionless variables are defined as:

$$X = \frac{B}{B_{max}}, \qquad Y = \frac{L}{K_M}, \qquad Z = \frac{U}{K_M}$$

And the dimensionless parameters are defined as:

$$\pi_1 = \frac{r}{k_{cat}}, \qquad \pi_2 = \frac{aK_M}{k_{cat}}, \qquad \pi_3 = \frac{B_{max}}{K_M}, \qquad \pi_4 = \frac{bB_{max}}{k_{cat}}$$

Steady-state Solution 1: For the steady-state solution where B = 0, the dimensionless variable solutions that follow are:

$$X = 0, \qquad Y = \frac{1}{\frac{\pi_2\pi_3}{\pi_1\pi_4} - 1}, \qquad Z = \frac{\pi_1}{\pi_2}$$

The resulting Jacobian, J, and eigen-values, λ, are:

$$J = \begin{bmatrix} 0 & 0 & 0 \\ -\pi_1\pi_4/\pi_2 & 0 & 0 \\ 0 & 0 & 0 \end{bmatrix}, \qquad \lambda = 0, 0, 0$$

Steady-state Solution 2: For the steady-state solution where B = B_{max}, the dimensionless variable solutions that follow are:

$$X = 1, \qquad Y = 0, \qquad Z = 0$$

The resulting Jacobian, J, and eigen-values, λ, are:

$$J = \begin{bmatrix} -\pi_1 & 0 & 0 \\ 0 & -\pi_3 & 0 \\ 0 & \pi_3 & -\pi_4 \end{bmatrix}, \qquad \lambda = -\pi_1, -\pi_3, -\pi_4$$

Since the eigen-values for steady-state solution 2 (i.e., B = B_{max}) are all negative, whereas the eigen-values for steady-state solution 1 (B = 0) are all equal to 0, we can conclude that steady-state solution 2 is stable and steady-state solution 1 is unstable.

To visualize these results, we rendered a three-dimensional quiver plot of the phase space for this model in MATLAB:

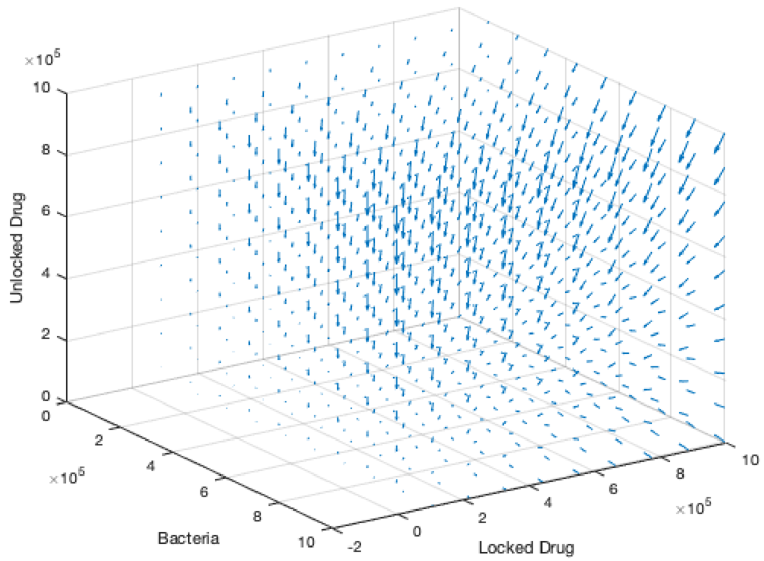

Based on the direction of the tangents, we can confirm that the B = B_{max} steady-state solution is stable.

Point 3. *"…Related to the comment above: the current model will have L=0 in the steady state since there is no source term for increasing L in equation 1.2, no?..."*

Response 3. It is true that one possible steady state includes the result $L_{ss} = 0$, however there is also another possible steady state where $L_{ss} > 0$. While there is no source term for increasing L in equation 1.2, the decay term is dependent on the bacteria population, *B* (i.e., when $B_{ss} = 0$, $L_{ss} > 0$). We see this result in our linear stability analysis (**Response 2**), where the steady state value of *L* is dependent on all system parameters in the solution where $B_{ss} = 0$.

Point 4. "…*Since the locked drug becomes unlocked, and the unlocked drug decays, the drug will eventually disappear and the cells will re-grow (since the population can never hit extinction in this model), right?..."*

Response 4. The bacteria population *can* hit extinction in this model, as observed multiple times (**Fig. 2c—h**, **Fig. 4b, d**). We know it is possible for the model to produce this result because we apply the 'non-negative' constraint to the MATLAB ODE solver; this constraint ensures that if a variable reaches a negative value, that variable will be clamped to 0. Further, as the reviewer mentions in **Point 2**, one possible steady-state solution includes the result where $B_{ss} = 0$ (i.e., the bacteria population hits extinction). Therefore, if the bacteria population reaches 0 before the drug has decayed, all other transition terms go to 0 because they are dependent on *B* and the system reaches a steady-state where $L_{ss} > 0$ and $U_{ss} > 0$ (**Response 2**).

However, the steady state described by the reviewer does occur when *L* and *U* reach 0 before *B* reaches 0. In this case, the drug will eventually disappear and the bacteria will regrow. This family of solutions represents the definition of prodrug escape used in this paper (**Figure 1**).

Point 5. "…*One option is to develop a stochastic version of the model that accounts for demographic noise and would allow for true extinction. But a much simpler approach is to consider adding a source (constant) term to Equation 1.2 that provides an influx of locked drug. Then you have a well-defined steady state solution that (presumably) bifurcates as r/kcat crosses some threshold. To compare to experiments, one option might be to choose that influx term so that the steady state of L matches the concentration you used in the experiment (or some estimate of its steady state concentration on the experimental timescale). One could then think of that model as valid on the experimental timescale, at least in the regime where the concentration of prodrug reaches some quasi steady state. On longer timescales, of course, the prodrug itself will decay, but then you are forced to study only the transient behavior of the model with numerics, as the steady state will be less interesting and always result in full growth and zero drug. If you add the source term and do the stability analysis, I think you'll find that the critical value of r/kcat depends on some of the other parameters through the (quasi) steady state value of L. My hunch is that the values you choose represent combinations of these parameters that could be predicted by this analysis…*"

Response 5. While we appreciate these recommendations and think they would be interesting to implement, these modifications are not necessary to implement because the model can already produce the result where the bacteria population reaches 0 (see **Response 4**). However, we did find that the critical *BAH* value depends on the ratio of the system parameters *a* and *b*. We found this by generating a range of linearly spaced values for parameters *a* and *b* and calculating the BAH_{crit} value for all permutations of *a* and *b*. Then, we plot the $\log_{10}$ of the ratio of *a* to *b* and observe a linear relationship between the ratio of *a* to *b* and the BAH_{crit} value. Given that the rest of the system parameters (r, k_{cat}, K_M, B_{max}) did not change the BAH_{crit} value, we can assume *a* and *b* are the only parameters other than initial conditions that may impact the BAH_{crit} value. We have added this analysis to **Fig. EV9**.

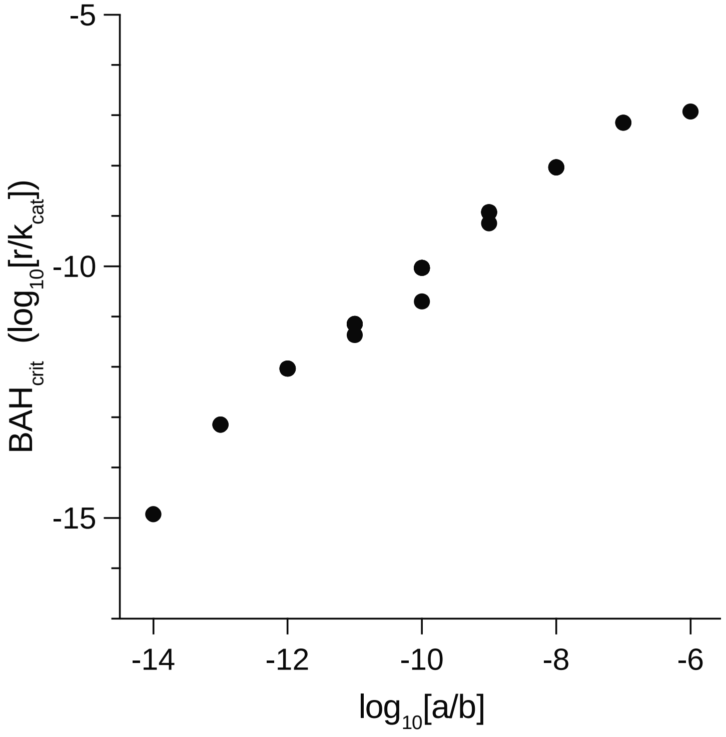

Point 6. *"…The feedback between population density and drug concentration seems to be driving many of the interesting results seen here. The authors may wish to comment on / connect their results to other known cases from the literature where similar feedback loops (driven by population density) contribute to the way bacteria respond to antibiotics. For example, similar feedback loops play a role in the classical inoculum effect (IE) as well as in adaptive therapies, which attempt to maintain drug sensitive populations as a competitive barrier to the emergence of resistance populations (these adaptive therapies have been studied in cancer, parasitic infections, and antibiotic resistance). These connections may open the work to a broader audience interested in antibiotic resistance and also raise interesting questions for future work (at least in my opinion). It might be nice to mention other studies that indicate how MIC alone is not always a good proxy for treatment efficacy, even for regular (not pro-) drugs. For example, metrics such as single-cell MIC (Jeff Gore lab) or other time dependent measures (L. You lab) have been developed for predicting treatment outcomes in cases where resistance is cooperative. I think the discussion could be broadened to include a bit more on this well-studied topic (and this does not detract from the novelty of the current study)."*

Response 6. We agree that adding these discussion points would be beneficial to the broad appeal of the manuscript. We find it interesting that there are analogous feedback loops in different systems that are similar to those observed in our work. For example, in the inoculum effect, the higher the population density being treated, the higher the concentration of antibiotic required to treat the bacteria (i.e., MIC). In a way, the feedback loop observed in our work is between the same two parameters, but deals with the first time-derivative of these parameters instead. In other words, the inoculum effect deals with the balance between the initial bacterial density (B_0) and initial unlocked drug concentration (U_0), whereas our system deals with the balance between the rate of change of bacterial density ($\frac{dB_0}{dt} \propto r$) and the rate of change of the unlocked drug concentration ($\frac{dU_0}{dt} \propto k_{cat}$). We have added citations to these other feedback loops in the discussion section:

"*We found that these prodrugs failed in conditions where bacterial growth outpaced the rate of prodrug activation, as predicted by our computational results. This feedback (i.e., feedback loop; Fig. 1b) between bacterial density and drug concentration is similar to the feedback between bacterial density and antibiotics in the inoculum effect, or the feedback between drug-insensitive cells and drug-sensitive cells in multi-drug adaptive therapies for cancer."*

We have also added text mentioning other studies that have developed metrics to complement the MIC, which does not always adequately predict treatment efficacy:

"Future iterations of this work and the BAH may result in a standardized quantitative design criteria that is specific to prodrugs. This is supported by the fact that even with non-prodrugs, metrics such as the single-cell MIC and others based on bacterial temporal dynamics have been developed for predicting treatment outcomes where MIC fails."

References

Al-Badr, A. A. and T. D. S. Ajarim (2018). Chapter One - Ganciclovir. Profiles of Drug Substances, Excipients and Related Methodology. H. G. Brittain, Academic Press. **43:** 1-208.

Allen, R. J. and B. Waclaw (2019). "Bacterial growth: a statistical physicist's guide." Reports on progress in physics. Physical Society (Great Britain) **82**(1): 016601-016601.

Edwards, D. I. (1993). "Nitroimidazole drugs-action and resistance mechanisms I. Mechanism of action." Journal of Antimicrobial Chemotherapy **31**(1): 9-20.

Eliopoulos, G. M. and P. Huovinen (2001). "Resistance to Trimethoprim-Sulfamethoxazole." Clinical Infectious Diseases **32**(11): 1608-1614.

Jenks, P. J., et al. (1999). "The role of the rdxA gene in the evolution of metronidazole resistance in Helicobacter pylori." Journal of Antimicrobial Chemotherapy **43**(6): 753-758.

Jin, D. J., et al. (2012). "Growth rate regulation in Escherichia coli." FEMS microbiology reviews **36**(2): 269-287.

Jubeh, B., et al. (2020). "Antibacterial Prodrugs to Overcome Bacterial Resistance." Molecules (Basel, Switzerland) **25**(7): 1543.

Kramer, R. A., et al. (2001). "Identification of essential acidic residues of outer membrane protease OmpT supports a novel active site." FEBS Lett **505**(3): 426-430.

Kramer, R. A., et al. (2000). "In vitro folding, purification and characterization of Escherichia coli outer membrane protease ompT." Eur J Biochem **267**(3): 885-893.

Le, V. V. H. and J. Rakonjac (2021). "Nitrofurans: Revival of an “old” drug class in the fight against antibiotic resistance." PLOS Pathogens **17**(7): e1009663.

Leiros, H. K., et al. (2004). "Structural basis of 5-nitroimidazole antibiotic resistance: the crystal structure of NimA from Deinococcus radiodurans." J Biol Chem **279**(53): 55840-55849.

Marais, A., et al. (2003). "Characterization of the genes rdxA and frxA involved in metronidazole resistance in Helicobacter pylori." Res Microbiol **154**(2): 137-144.

McKeegan, K. S., et al. (2002). "Microbial and viral drug resistance mechanisms." Trends Microbiol **10**(10 Suppl): S8-14.

Metcalfe, C., et al. (2008). "The tuberculosis prodrug isoniazid bound to activating peroxidases." J Biol Chem **283**(10): 6193-6200.

Rautio, J., et al. (2018). "The expanding role of prodrugs in contemporary drug design and development." Nature Reviews Drug Discovery **17**: 559.

Thomassin, J.-L., et al. (2012). "OmpT outer membrane proteases of enterohemorrhagic and enteropathogenic Escherichia coli contribute differently to the degradation of human LL-37." Infection and immunity **80**(2): 483-492.

Weissman, J. L., et al. (2021). "Estimating maximal microbial growth rates from cultures, metagenomes, and single cells via codon usage patterns." Proceedings of the National Academy of Sciences **118**(12): e2016810118.

1st Revision - Editorial Decision 9th Dec 2021

Thank you for sending us your revised manuscript. We have now heard back from reviewer #2. As you will see below, they are satisfied with the modifications made and are supportive for publication. Unfortunately, after several reminders we have not received a report from reviewer #3. However, we have evaluated your answers to their concerns and think that the performed revisions have satisfactorily addressed the issues raised by reviewer #3. As such, I am glad to inform you that we can soon proceed with formally accepting the study for publication, pending some minor editorial issues listed below. We would ask you to address these issues in a minor revision.

REFEREE REPORTS

Reviewer #2:

The authors have successfully addressed my previous concerns and questions through clarifying discussions in the text and by providing additional experimental data. I support publication of this manuscript in MSB and I believe that this study is a valuable addition to the field of antibacterial research.

2nd Authors' Response to Reviewers 15th Dec 2021

The authors have made all requested editorial changes.

ACCEPTED **17th Dec 2021**

Thank you again for sending us your revised manuscript. We are now satisfied with the modifications made and I am pleased to inform you that your paper has been accepted for publication.

EMBO PRESS

YOU MUST COMPLETE ALL CELLS WITH A PINK BACKGROUND ➔

PLEASE NOTE THAT THIS CHECKLIST WILL BE PUBLISHED ALONGSIDE YOUR PAPER

Corresponding Author Name: Gabriel Kwong

Journal Submitted to: Molecular Systems Biology

Manuscript Number: MSB-2021-10495

Reporting Checklist For Life Sciences Articles (Rev. June 2017)

This checklist is used to ensure good reporting standards and to improve the reproducibility of published results. These guidelines are consistent with the Principles and Guidelines for Reporting Preclinical Research issued by the NIH in 2014. Please follow the journal's authorship guidelines in preparing your manuscript.

A- Figures

1. Data

The data shown in figures should satisfy the following conditions:

- the data were obtained and processed according to the field's best practice and are presented to reflect the results of the experiments in an accurate and unbiased manner.
- figure panels include only data points, measurements or observations that can be compared to each other in a scientifically meaningful way.
- graphs include clearly labeled error bars for independent experiments and sample sizes. Unless justified, error bars should not be shown for technical replicates.
- if n< 5, the individual data points from each experiment should be plotted and any statistical test employed should be justified
- Source Data should be included to report the data underlying graphs. Please follow the guidelines set out in the author ship guidelines on Data Presentation.

2. Captions

Each figure caption should contain the following information, for each panel where they are relevant:

- a specification of the experimental system investigated (eg cell line, species name).
- the assay(s) and method(s) used to carry out the reported observations and measurements
- an explicit mention of the biological and chemical entity(ies) that are being measured.
- an explicit mention of the biological and chemical entity(ies) that are altered/varied/perturbed in a controlled manner.

- the exact sample size (n) for each experimental group/condition, given as a number, not a range;
- a description of the sample collection allowing the reader to understand whether the samples represent technical or biological replicates (including how many animals, litters, cultures, etc.).
- a statement of how many times the experiment shown was independently replicated in the laboratory.
- definitions of statistical methods and measures:
  - common tests, such as t-test (please specify whether paired vs. unpaired), simple χ2 tests, Wilcoxon and Mann-Whitney tests, can be unambiguously identified by name only, but more complex techniques should be described in the methods section;
  - are tests one-sided or two-sided?
  - are there adjustments for multiple comparisons?
  - exact statistical test results, e.g., P values = x but not P values < x;
  - definition of 'center values' as median or average;
  - definition of error bars as s.d. or s.e.m.

Any descriptions too long for the figure legend should be included in the methods section and/or with the source data.

In the pink boxes below, please ensure that the answers to the following questions are reported in the manuscript itself. Every question should be answered. If the question is not relevant to your research, please write NA (non applicable). We encourage you to include a specific subsection in the methods section for statistics, reagents, animal models and human subjects.

USEFUL LINKS FOR COMPLETING THIS FORM

http://www.antibodypedia.com
http://1degreebio.org
http://www.equator-network.org/reporting-guidelines/improving-bioscience-research-report

http://grants.nih.gov/grants/olaw/olaw.htm
http://www.mrc.ac.uk/Ourresearch/Ethicsresearchguidance/Useofanimals/index.htm
http://ClinicalTrials.gov
http://www.consort-statement.org
http://www.consort-statement.org/checklists/view/32-consort/66-title

http://www.equator-network.org/reporting-guidelines/reporting-recommendations-for-tumo

http://datadryad.org

http://figshare.com

http://www.ncbi.nlm.nih.gov/gap

http://www.ebi.ac.uk/ega

http://biomodels.net/

http://biomodels.net/miriam/
http://jjj.biochem.sun.ac.za
https://osp.od.nih.gov/biosafety-biosecurity-and-emerging-biotechnology/
http://www.selectagents.gov/

B- Statistics and general methods

Please fill out these boxes ➔ (Do not worry if you cannot see all your text once you press return)

1.a. How was the sample size chosen to ensure adequate power to detect a pre-specified effect size?	For in vitro experiments, sample size of n = 3 - 6 was used as a conservative approach based on estimates of effect size from preliminary experiments to reach a statistical power equal to or greater than 0.8.
1.b. For animal studies, include a statement about sample size estimate even if no statistical methods were used.	NA
2. Describe inclusion/exclusion criteria if samples or animals were excluded from the analysis. Were the criteria pre-established?	The only exclusion criteria used was in bacterial viability experiments when plated colonies were smeared, contaminated, or malformed in a way that made it impossible to count individual colonies. This criteria was pre-established.
3. Were any steps taken to minimize the effects of subjective bias when allocating animals/samples to treatment (e.g. randomization procedure)? If yes, please describe.	All bacteria were randomly pulled from a well-mixed solution.
For animal studies, include a statement about randomization even if no randomization was used.	NA
4.a. Were any steps taken to minimize the effects of subjective bias during group allocation or/and when assessing results (e.g. blinding of the investigator)? If yes please describe.	All samples were placed in wells according to group and notated in lab notebook. These labels were hidden during the collection of data during the experiment, and were not revealed until after data was collected. These labels were used for plot creation, at which point the labels were known. Statistical analysis was performed automatically by graphing software so during statistical anlysis the labels were blinded to the researcher.
4.b. For animal studies, include a statement about blinding even if no blinding was done	NA
5. For every figure, are statistical tests justified as appropriate?	Yes
Do the data meet the assumptions of the tests (e.g., normal distribution)? Describe any methods used to assess it.	Yes
Is there an estimate of variation within each group of data?	Yes

Is the variance similar between the groups that are being statistically compared?	Yes

C- Reagents

6. To show that antibodies were profiled for use in the system under study (assay and species), provide a citation, catalog number and/or clone number, supplementary information or reference to an antibody validation profile. e.g., Antibodypedia (see link list at top right), 1DegreeBio (see link list at top right).	NA
7. Identify the source of cell lines and report if they were recently authenticated (e.g., by STR profiling) and tested for mycoplasma contamination.	NA

* for all hyperlinks, please see the table at the top right of the document

D- Animal Models

8. Report species, strain, gender, age of animals and genetic modification status where applicable. Please detail housing and husbandry conditions and the source of animals.	NA
9. For experiments involving live vertebrates, include a statement of compliance with ethical regulations and identify the committee(s) approving the experiments.	NA
10. We recommend consulting the ARRIVE guidelines (see link list at top right) (PLoS Biol. 8(6), e1000412, 2010) to ensure that other relevant aspects of animal studies are adequately reported. See author guidelines, under 'Reporting Guidelines'. See also: NIH (see link list at top right) and MRC (see link list at top right) recommendations. Please confirm compliance.	NA

E- Human Subjects

11. Identify the committee(s) approving the study protocol.	NA
12. Include a statement confirming that informed consent was obtained from all subjects and that the experiments conformed to the principles set out in the WMA Declaration of Helsinki and the Department of Health and Human Services Belmont Report.	NA
13. For publication of patient photos, include a statement confirming that consent to publish was obtained.	NA
14. Report any restrictions on the availability (and/or on the use) of human data or samples.	NA
15. Report the clinical trial registration number (at ClinicalTrials.gov or equivalent), where applicable.	NA
16. For phase II and III randomized controlled trials, please refer to the CONSORT flow diagram (see link list at top right) and submit the CONSORT checklist (see link list at top right) with your submission. See author guidelines, under 'Reporting Guidelines'. Please confirm you have submitted this list.	NA
17. For tumor marker prognostic studies, we recommend that you follow the REMARK reporting guidelines (see link list at top right). See author guidelines, under 'Reporting Guidelines'. Please confirm you have followed these guidelines.	NA

F- Data Accessibility

18: Provide a "Data Availability" section at the end of the Materials & Methods, listing the accession codes for data generated in this study and deposited in a public database (e.g. RNA-Seq data: Gene Expression Omnibus GSE39462, Proteomics data: PRIDE PXD000208 etc.) Please refer to our author guidelines for 'Data Deposition'.<br><br>Data deposition in a public repository is mandatory for:<br>a. Protein, DNA and RNA sequences<br>b. Macromolecular structures<br>c. Crystallographic data for small molecules<br>d. Functional genomics data<br>e. Proteomics and molecular interactions	The authors declare that the data supporting the findings of this study are available within the paper and its supplementary information files. The authors declare that the code supporting the findings of this study is available within the supplementary information files and at https://github.com/brandon-holt/bacterial-advantage-heuristic
19. Deposition is strongly recommended for any datasets that are central and integral to the study; please consider the journal's data policy. If no structured public repository exists for a given data type, we encourage the provision of datasets in the manuscript as a Supplementary Document (see author guidelines under 'Expanded View' or in unstructured repositories such as Dryad (see link list at top right) or Figshare (see link list at top right).	NA
20. Access to human clinical and genomic datasets should be provided with as few restrictions as possible while respecting ethical obligations to the patients and relevant medical and legal issues. If practically possible and compatible with the individual consent agreement used in the study, such data should be deposited in one of the major public access-controlled repositories such as dbGAP (see link list at top right) or EGA (see link list at top right).	NA
21. Computational models that are central and integral to a study should be shared without restrictions and provided in a machine-readable form. The relevant accession numbers or links should be provided. When possible, standardized format (SBML, CellML) should be used instead of scripts (e.g. MATLAB). Authors are strongly encouraged to follow the MIRIAM guidelines (see link list at top right) and deposit their model in a public database such as Biomodels (see link list at top right) or JWS Online (see link list at top right). If computer source code is provided with the paper, it should be deposited in a public repository or included in supplementary information.	All computational models are provided in a public GitHub repository.

G- Dual use research of concern

22. Could your study fall under dual use research restrictions? Please check biosecurity documents (see link list at top right) and list of select agents and toxins (APHIS/CDC) (see link list at top right). According to our biosecurity guidelines, provide a statement only if it could.	NA
